# Mixing alters the lytic activity of viruses in the dark ocean

**DOI:** 10.1002/ecy.2135

**Published:** 2018-02-06

**Authors:** Christian Winter, Nicole Köstner, Carl‐Philip Kruspe, Damaris Urban, Simone Muck, Thomas Reinthaler, Gerhard J. Herndl

**Affiliations:** ^1^ Department of Limnology and Bio‐Oceanography Center of Ecology University of Vienna Althanstrasse 14 Vienna 1090 Austria

**Keywords:** deep ocean, frequency of infected cells, microbial loop, mixing, prokaryotes, viral production, viruses

## Abstract

In aquatic habitats, viral lysis of prokaryotic cells lowers the overall efficiency of the microbial loop, by which dissolved organic carbon is transfered to higher trophic levels. Mixing of water masses in the dark ocean occurs on a global scale and may have far reaching consequences for the different prokaryotic and virus communities found in these waters by altering the environmental conditions these communities experience. We hypothesize that mixing of deep ocean water masses enhances the lytic activity of viruses infecting prokaryotes. To address this hypothesis, major deep‐sea water masses of the Atlantic Ocean such as North Atlantic Deep Water, Mediterranean Sea Overflow Water, Antarctic Intermediate Water, and Antarctic Bottom Water were sampled at five locations. Prokaryotic cells from these samples were collected by filtration and subsequently incubated in virus‐reduced water from either the same (control) or a different water mass (transplantation treatment). Additionally, mixtures of prokaryotes obtained from two different water masses were incubated in a mixture of virus‐reduced water from the same water masses (control) or in virus‐reduced water from the source water masses separately (mixing treatments). Pronounced differences in productivity‐related parameters (prokaryotic leucine incorporation, prokaryotic and viral abundance) between water masses caused strong changes in viral lysis of prokaryotes. Often, mixing of water masses increased viral lysis of prokaryotes, indicating that lysogenic viruses were induced into the lytic cycle. Mixing‐induced changes in viral lysis had a strong effect on the community composition of prokaryotes and viruses. Our data show that mixing of deep‐sea water masses alters levels of viral lysis of prokaryotes and in many cases weakens the efficiency of the microbial loop by enhancing the recycling of organic carbon in the deep ocean.

## Introduction

The composition of prokaryotic (members of the domains *Bacteria* and *Archaea*, no phylogenetic relationship is implied) and virus communities changes throughout the oceanic water column (Galand et al. 2010, Agogué et al. 2011, Winter et al. 2013, Hurwitz et al. 2014) because the ocean is vertically structured. For example, the sunlit surface layer is separated by a thermocline from the deep ocean (>200 m depth). Given that temperature and the concentration of dissolved salts (salinity) determine the density of water, the sunlit water above the thermocline is warmer and, thus, lighter compared to the water below it. Thus, the deep ocean is separated from the atmosphere by a thin layer of lighter and warmer surface water and is considered to be a stable ecosystem, where changes are presumed to occur on the scale of years to decades (e.g., Béthoux et al. 1990) rather than weeks to months. Nevertheless, prokaryotic community composition in the deep‐sea can be as dynamic as in surface waters and may even exhibit seasonality (Fuhrman et al. 2006, Winter et al. 2009a *,* b, 2010). The deep ocean itself is vertically‐structured due to fine‐grained differences in temperature and salinity resulting in subtle density differences that give rise to defined water masses (Emery 2001) and harbor distinct prokaryotic and virus communities (Galand et al. 2010, Agogué et al. 2011, Winter et al. 2013, Hurwitz et al. 2014).

In the North and Subtropical Atlantic Ocean, two relevant intermediate (500–1,800 m depth) and two deep (1,800 m to bottom) water masses are found (van Aken 2000a *,* b, Emery 2001). North Atlantic Deep Water (NADW), the major deep water mass, forms between Greenland and Iceland and flows southwards throughout the Atlantic Ocean. NADW mixes with Antarctic Intermediate Water (AAIW) and in the area of the Strait of Gibraltar with Mediterranean Sea Outflow Water (MSOW). AAIW originates in the Antarctic Polar Front and flows northwards on top of NADW. Antarctic Bottom Water (AABW) constitutes the densest water in the ocean and is formed through surface water cooling in the Weddel and Ross Seas. In the Atlantic Ocean, AABW flows northwards at the bottom of the western basin and enters the eastern basin through fracture zones in the Mid‐Atlantic Ridge at the equator and 10° N. Diffusive mixing of AABW and NADW occurs wherever both water masses are present. Additionally, ocean floor topography facilitates turbulent mixing of AABW with NADW due to the passage of water through narrow clefts and over ridges (Polzin et al. 1996, Ferron et al. 1998, Bryden and Nurser 2003, St Laurent and Thurnherr 2007, Lozovatsky et al. 2008, MacKinnon et al. 2008).

Prokaryotes make dissolved organic carbon (DOC) available to higher trophic levels by consuming up to ~50% of the available DOC (microbial loop; Pomeroy 1974, Azam et al. 1983). Viral lysis transforms cells, i.e. particulate organic matter, into dissolved organic matter, thus, reducing the efficiency of the microbial loop (viral shunt; Fuhrman 1999, Wilhelm and Suttle 1999, Jover et al. 2014). In the ocean, viruses are by far the most abundant biological entity and behind prokaryotes constitute the second largest pool of biomass (Suttle 2007). Viruses are obligate parasites and lack the ability for active movement making finding the right host organism a stochastic process that is abundance‐dependent (Murray and Jackson 1992). Thus, the low prokaryotic abundance (~10^4^–10^5^ mL^−1^; e.g., Ortmann and Suttle 2005, Magagnini et al. 2007, Winter et al. 2009a) in the deep ocean may restrict viral infection and production rates. However, the high numbers of viruses found in the deep (~10^5^–10^7^ mL^−1^; e.g., Mei and Danovaro 2004, Magagnini et al. 2007, Winter et al. 2009a) suggest that virus production has to occur in order to balance the losses.

Viruses infecting prokaryotes are considered to be species‐specific (but also see Sullivan et al. 2003, Holmfeldt et al. 2007). Consequently, different host communities will result in different actively‐reproducing virus communities (e.g., Larsen et al. 2004, Hewson and Fuhrman 2007, Winter and Weinbauer 2010). Viruses may also influence their host community by selectively killing the winners in the competition for nutrients (“killing the winner” hypothesis; Thingstad 2000, Rodriguez‐Valera et al. 2009, Winter et al. 2010), considered to be a key mechanism in maintaining prokaryotic richness. Most viruses infecting prokaryotes are either lytic or lysogenic. Lytic viruses initiate virus production shortly after successful infection and kill their host by lysis, releasing progeny virus particles into the environment. Lysogenic viruses may either initiate the lytic cycle or integrate their genome into the host's genome and remain dormant. These prophages form a lasting and symbiotic relationship with their host (e.g., Chen et al. 2005) until the lytic cycle is induced. Induction of lysogens may be due to the presence of inducing agents (e.g., ultraviolet radiation) or may also happen in the absence of external triggers (spontaneous prophage induction, e.g., Nanda et al. 2015). The best studied mechanism for inducing many lysogenic viruses involves DNA damage sustained by the host due to the exposure to ultraviolet radiation (Jiang and Paul 1996, Weinbauer and Suttle 1999). DNA damage in prokaryotes leads to the activation of the RecA‐dependent SOS response (Michel 2005) and also facilitates the degradation of certain phage repressor proteins, leading to the induction of lysogenic viruses. Nevertheless, several RecA‐independent induction mechanisms have been described in model systems (e.g., Rozanov et al. 1998, Shkilnyi and Koudelka 2007, Ghosh et al. 2009, Erez et al. 2017). Also, the level of lysogeny in viral communities at the surface may be influenced by environmental parameters such as temperature, trophic conditions, or drastic changes in salinity (e.g., Williamson and Paul 2004, 2006, Cissoko et al. 2008, Bettarel et al. 2011). In general, lysogenic viruses appear to be more common in environments with low host abundance and/or activity such as the deep sea, whereas lytic viruses are expected to thrive especially at moderate to high host abundances and/or activities (Weinbauer et al. 2003, Paul 2008). However, the environmental factors influencing the lysogenic‐lytic switch are poorly understood, certainly so in the deep sea (Paul 2008).

We hypothesize that mixing of deep ocean water masses enhances the lytic activity of viruses infecting prokaryotes due to enhanced growth of a subset of the prokaryotic community, well adapted to the altered environmental conditions found in the resulting mix of the two parent water masses, thus, producing viruses faster. We tested this hypothesis in experiments based on a factorial design. The frequency of infected prokaryotic cells (FIC, percentage of prokaryotes infected by viruses) and viral production (VP, rate at which viruses are produced) were determined as a measure of viral lytic activity using a virus‐dilution approach to prevent new viral infections during the experiments (Winter et al. 2004, Köstner et al. 2017). We also wanted to know whether potential changes in viral lytic activity as a consequence of mixing would affect the community composition of prokaryotes and viruses as detected by fingerprinting approaches. In this study, the environmental conditions are changed by mixing samples of two parent water masses, mimicking mixing in situ, instead of experimentally manipulating a specific parameter or group of parameters. This is due to the lack of information on specific parameters with the potential to alter levels of lysogeny in the deep ocean. Yet, based on previous studies conducted at the surface (Williamson and Paul 2006, Cissoko et al. 2008, Bettarel et al. 2011), the experimental results were related to salinity and temperature of the two parent water masses (Emery 2001). Additionally, differences in trophic conditions have been implicated to alter the percentage of lysogenically infected prokaryotic cells at the surface (Williamson and Paul 2004, 2006). Thus, prokaryotic leucine incorporation as a measure for prokaryotic heterotrophic activity together with prokaryotic and viral abundance were used as a proxy for the trophic conditions of the parent water masses (Wommack and Colwell 2000, Finke et al. 2017).

## Methods

### Sampling and filtrations

**Figure 1:**
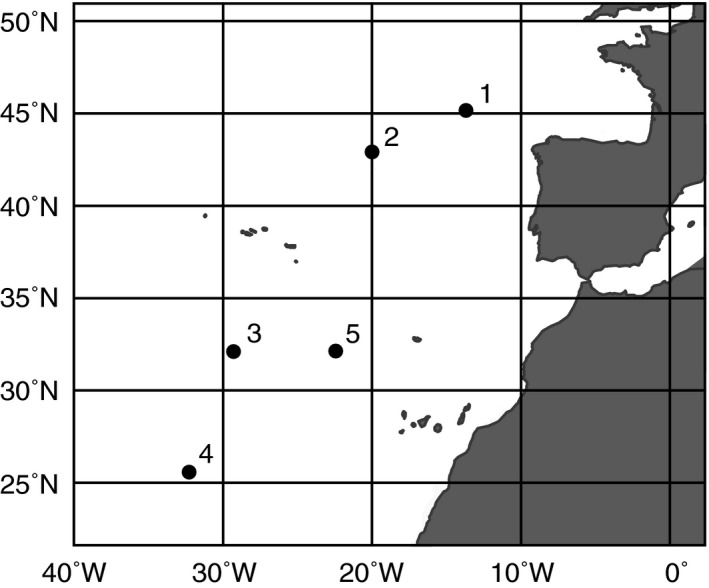
Map of the sampling area. The figure shows the location of the 5 sampling stations in the Atlantic Ocean.

Water samples were retrieved at five stations during a cruise in the Atlantic Ocean from October–November 2011. At each station (Fig. 1), two samples (160 L each) were retrieved using Niskin bottles (OceanTest Equipment Inc., Fort Lauderdale, Florida, USA) mounted on a rosette sampler also carrying sensors for conductivity, temperature, and depth (all from SeaBird Electronics, Washington, USA). Water masses were identified on plots of salinity vs. potential temperature (the temperature of a body of water when it would be raised from depth to the surface such that its volume is allowed to adjust to the lower pressure at the surface; Emery 2001; Appendix S2: Fig. S1). At every station, one water sample always corresponded to NADW (2,750 m depth), the major deep‐sea water mass in the sampling area. The second water sample at station 1 corresponded to MSOW (900 m depth), at stations 2–3 to AAIW (station 2: 1,300 m depth; station 3: 1,700 m depth), and at stations 4–5 to AABW (5,000 m depth). Immediately after sampling, subsamples to determine the productivity‐related biological parameters prokaryotic and viral abundance as well as prokaryotic heterotrophic activity by ^3^H‐leucine incorporation were taken and processed as described herein.

Subsequently, tangential‐flow filtration devices were used to obtain a prokaryotic concentrate and a virus‐free ultrafiltrate from each water sample to be used in the experiments. Specifically, prokaryotes were concentrated using a tangential‐flow filtration unit with a pore‐size of 0.22 μm (Pellicon 2 System, filter module P2GVPPC05, Durapore, Merck Millipore, Darmstadt, Germany) until the volume of the concentrate reached 700 mL. Subsequently, viruses contained in the filtrate of the first filtration step were removed by a tangential‐flow filtration unit with a molecular weight cut‐off of 100 kDa (PTHK Prep/Scale TFF, polyethersulfone, Merck Millipore). To avoid cross‐contamination, both filtration devices were cleaned with 70% ethanol, rinsed with 10 L of Milli‐Q water and up to 5 L of sample before collecting the prokaryotic concentrate and virus‐free ultrafiltrate.

### Experimental set‐up and sub‐sampling

The experiments consisted of seven duplicate treatments per sampling station using acid‐cleaned 20 L carboys (Nalgene, Rochester, New York, USA) as incubation vessels. The first four treatments consisted of adding equal volumes of prokaryotic concentrate to virus‐free ultrafiltrate from the same (control) or the other (transplantation) water mass (Appendix S2: Fig. S2a). The final three treatments were mixing treatments with equal volumes of prokaryotic concentrates from both water masses, added to ultrafiltrate from the first, the second, or an equal mixture of both water masses (control; Appendix S2: Fig. S2b). The final volume of each treatment was 20 L and the experiments were incubated in the dark at 2–6°C (depending on the sampled water masses; Appendix S2: Fig. S1) for 72 h.

Prokaryotic and viral abundance was determined at the start of the incubation and every 4–5 h as described below in order to calculate FIC and VP (Winter et al. 2004, Köstner et al. 2017). At the onset of the experiments and every 24 h, subsamples were taken to obtain fingerprints of the bacterial, archaeal, and viral community composition to detect possible changes in the host and corresponding virus community in response to the experimental treatments as follows. Prokaryotes and viruses in 1 L subsamples were concentrated by sequential tangential‐flow filtration (Vivaflow 200; prokaryotes: pore‐size of 0.22 μm, viruses: molecular weight cut‐off of 100 kDa; Sartorius, Göttingen, Germany) until the concentrates reached a volume of 50 mL. Subsequently, prokaryotes were collected by filtering the concentrate over a membrane filter with a pore‐size of 0.22 μm (Durapore, 47 mm diameter, GVWP, Merck Millipore). The filters were flash‐frozen and stored at −80°C until analyses. The viral concentrates were flash‐frozen directly and stored at −80°C. Upon thawing, viral concentrates were filtered over syringe filters with a pore‐size of 0.22 μm (Acrodisc, 25 mm diameter, Supor membrane, Pall Corporation, Ann Arbor, Michigan, USA) to ascertain that the concentrates were free of any residual cells and other large particles. Subsequently, viruses were further concentrated using centrifugal filters with a molecular weight cut‐off of 100 kDa (Amicon Ultra‐15, Merck Millipore). The final viral concentrate had a volume of 200 μL and was used directly for extracting viral nucleic acids as described below.

### Determination of prokaryotic and viral abundance

Samples for the enumeration of prokaryotes and viruses (1.8 mL) were fixed immediately with glutaraldehyde (0.5% final concentration) at room temperature for 10 min, flash‐frozen in liquid nitrogen, and stored at −80°C until analyses. Enumeration of prokaryotic cells and viruses after staining with SYBR Green I (Thermo Fisher Scientific, Ulm, Germany) was performed on a FACSAria II flow cytometer (BD Biosciences, Mississauga, Ontario, Canada) as previously described (Marie et al. 1999, Brussaard et al. 2010). The abundance of prokaryotes and viruses is given as the average of duplicate (experimental samples) or triplicate (in situ samples) measurements.

### Determination of prokaryotic leucine incorporation

Prokaryotic leucine incorporation as a measure of prokaryotic heterotrophic activity was used together with prokaryotic and viral abundance as a proxy for the trophic conditions, because changing trophic conditions have been implicated in altering levels of lysogeny in aquatic surface habitats (Williamson and Paul 2004, 2006). Prokaryotic heterotrophic activity of unfiltered seawater and at the end of the incubation period from each experimental treatment was measured by ^3^H‐leucine incorporation (specific activity: 120 Ci/mmol; final concentration 5–10 nmol/L, PerkinElmer New England Nuclear, Waltham, Massachusetts, USA) as previously described (Reinthaler et al. 2010). Cell‐specific prokaryotic leucine incorporation was calculated by dividing prokaryotic leucine incorporation through prokaryotic abundance. Leucine incorporation is given as the mean of triplicate incubations corrected for the mean of two blanks.

### Estimation of the frequency of infected cells and viral production

Frequency of infected cells (FIC) and viral production (VP) were estimated from the temporal development of viral abundance in the experimental incubations, adhering to the requirements of the virus dilution approach (Winter et al. 2004, Köstner et al. 2017; Appendix S3). For that, viruses were removed by tangential‐flow filtration during set‐up of the experiments (see above). Because virus infection is density‐dependent (Murray and Jackson 1992), increasing viral abundance in the experimental incubations is a result of viral infections from before the sample was taken as new viral infections are prevented due to the much lower viral abundance compared to in situ conditions. For each experimental incubation, FIC and VP were calculated after 32 h and 72 h. In order to calculate FIC, we assumed a constant burst‐size of 30 viruses released per lysed prokaryotic cell (Appendix S3: Eq. S1; Weinbauer et al. 2003, Winter et al. 2004, Parada et al. 2006). The controls where prokaryotes obtained from a single water mass were incubated in ultrafiltrate from the same water mass for 32 h are akin to normal virus‐dilution incubations used to determine FIC and VP (Winter et al. 2004, Köstner et al. 2017). These VP data were corrected for differences between prokaryotic abundance at the onset of the experiments and in situ prokaryotic abundance.

### Determination of bacterial and archaeal community fingerprints by terminal restriction fragment length polymorphism (T‐RFLP) analysis

#### Nucleic acid extraction and PCR amplification

Prokaryotic nucleic acids were extracted from the filters using an UltraClean Soil DNA Isolation Kit (alternative lysis method: heating twice to 70°C, Mo Bio Laboratories Inc., Carlsbad, California, USA). Fragments of the 16S rRNA gene were amplified by PCR using primers 27F (5′‐AGA GTT TGA TCC TGG CTC AG‐3′) and 1492R (5′‐GGT TAC CTT GTT ACG ACT T‐3′; Lane 1991) for *Bacteria* and 21F (5′‐TTC CGG TTG ATC CYG CCG GA‐3′) and 958R (5′‐YCC GGC GTT GAM TCC AAT T‐3′; DeLong 1992) for *Archaea*. Primers 27F and 21F were fluorescently labeled on the 5′‐end with 6‐carboxyfluorescein (Thermo Fisher Scientific) and primers 1492R and 958R were 5′‐end labeled with the fluorescent dye VIC (Applied Biosystems UK, Warrington, UK). Each 50 μL PCR reaction contained 5 μL 10× reaction buffer (100 mmol/L Tris‐HCL [pH 8.8], 500 mmol/L KCl, 0.8% [v/v] glycerol), 4 μL MgCl_2_ (25 mmol/L, final concentration 2 mmol/L), 1.25 μL dNTP mix (10 mmol/L each, Cat. No. 10297‐018, Thermo Fisher Scientific), 2.5 μL of each 10 μmol/L forward and reverse primer solution, 0.25 μL Taq DNA polymerase (recombinant, 5 units/μL, Cat. No. 10342‐178, Thermo Fisher Scientific). The volume of prokaryotic nucleic acid extract (1–5 μL) added as template to the PCR reactions was adjusted to obtain adequate concentrations of PCR products. Cycling (Mastercycler pro S, Eppendorf, Hamburg, Germany) started with an initial denaturation at 95°C for 5 min, followed by 30 cycles of 95°C for 1 min, 55°C for 1 min, 72°C for 1 min. The PCR amplifications finished with a final elongation step at 72°C for 30 min (Janse et al. 2004) and a hold at 4°C. PCR products were purified (PCRExtract Mini Kit, Cat. No. 2300610, 5Prime, Hilden, Germany) and sized by standard agarose gel electrophoresis. The final volume of PCR fragments was 50 μL in 10 mmol/L Tris‐HCl (pH 8.5).

#### Restriction digestion

Each 15 μL restriction digest contained 1.5 μL 10× CutSmart buffer (500 mmol/L K‐acetate, 200 mmol/L Tris‐acetate, 100 mmol/L Mg‐acetate, 1 mg/mL BSA, pH 7.9) and 0.5 μL of restriction enzyme *Hha*I (20,000 units/mL; Cat. No. R0139S, both from New England BioLabs, Ipswich, Massachusetts, USA). The amount of PCR products added to the restriction digests (1–12 μL) was standardized using a NanoDrop 2000 spectrophotometer (Thermo Fisher Scientific). The digests were incubated at 37°C for 12 h followed by 65°C for 20 min to inactivate the enzyme.

#### Data collection and analysis

One μL of restriction fragments was denatured at 95°C for 3 min in 10 μL Hi‐Di formamide (Cat. No. 4311320) containing 0.3 μL GeneScan 1200 LIZ size standard (Cat. No. 4379950; both from Applied Biosystems‐Thermo Fisher Scientific). Sizing of fluorescently‐labeled restriction fragments was performed on an automated capillary sequencer (Applied Biosystems 3130XL, Thermo Fisher Scientific). Peak patterns were analyzed with PeakScanner software (version 1.0, Thermo Fisher Scientific) and translated into a binary data matrix (presence‐absence).

### Determination of virus community fingerprints by randomly amplified polymorphic DNA polymerase chain reaction (RAPD‐PCR) analysis

#### Viral nucleic acid extraction and PCR amplification

Nucleic acids were extracted from 100 μL of the final viral concentrate (see above) using a QIAmp MinElute Virus Spin Kit (including carrier RNA, Cat. No. 57704, QIAGEN GmbH, Hilden, Germany). The extracts had a final volume of 30 μL in AVE buffer (QIAGEN). Every sample was subjected to two PCR reactions with either primer OPA‐13 (5′‐CAG CAC CCA C‐3′) or CRA‐22 (5′‐CCG CAG CCA A‐3′; Neilan 1995, both from Thermo Fisher Scientific). Each 50 μL PCR reaction contained 5 μL 10× reaction buffer (100 mmol/L Tris‐HCl [pH 8.4], 500 mmol/L KCl), 1.5 μL MgCl_2_ (50 mmol/L, final concentration 1.5 mmol/L), 1 μL dNTP mix (10 mmol/L each, Cat. No. 10297‐018, Thermo Fisher Scientific), either 10 μL of a 10 μmol/L solution of primer OPA‐13 or 5 μL of a 10 μmol/L solution of primer CRA‐22, and 0.4 μL of Platinum Taq DNA Polymerase (5 units/μL, Cat. No. 10966‐026, Thermo Fisher Scientific). The amount of template for PCR amplification (0.5–5 μL) was adjusted to obtain adequate concentrations of PCR products. Cycling (Master cycler S, Eppendorf, Hamburg, Germany) started with an initial denaturation at 94°C for 10 min, followed by 30 cycles of 94°C for 30 s, 35°C for 3 min, 72°C for 1 min. A final elongation step at 72°C for 30 min was performed to avoid the formation of artifacts (Janse et al. 2004) followed by a final hold at 4°C.

#### Data collection and analysis

The concentration of PCR products was adjusted to similar levels based on comparison with a molecular mass standard (SmartLadder, Cat. No. MW‐1700‐10, Eurogentec, Liège, Belgium). Sizing of PCR products was performed in comparison to size standards (SmartLadder, Cat. No. MW‐1700‐10, Eurogentec, Liège, Belgium; GeneRuler, Cat. No. SM0311, Thermo Fisher Scientific) on 2.5% agarose gels run in 1× TBE buffer (89 mmol/L Tris‐HCl [pH 8.3], 89 mmol/L boric acid, 2 mmol/L EDTA) at 3 V/cm electrode distance for 135 min. Subsequently, the gels were stained with SYBR Gold (1:10,000 dilution of commercial stock solution, Thermo Fisher Scientific) for 30 min and electronic gel images were acquired using a Gel Doc XR+ gel documentation system (Bio‐Rad, Hercules, California, USA). Images were analyzed using the software Quantity One (version 4.6.8, Bio‐Rad) and the banding patterns were translated into a binary data matrix (presence‐absence; Winter and Weinbauer 2010).

### Network analyses based on co‐occurrence patterns of prokaryotes with viruses

The patterns of peaks (T‐RFLP) and bands (RAPD‐PCR; Data S1) obtained by the used fingerprinting techniques do not afford a full characterization of all types of *Bacteria*,* Archaea*, and viruses present in the samples. Thus, individual bands and peaks are interpreted as operational taxonomic units (OTUs), not specific types of prokaryotes or viruses. Bacterial and archaeal fingerprints based on forward and reverse primers as well as viral fingerprints from primers OPA‐13 and CRA‐22 were concatenated to yield three data sets (*Bacteria*,* Archaea*, viruses). Subsequently, the simultaneous detection of specific prokaryotic and viral OTUs (co‐occurrence) was recorded for each duplicate treatment and time point. These co‐occurrence patterns were translated into network graphs, where prokaryotic and viral OTUs were represented by a node (vertex) and the co‐occurrence between prokaryotic and viral OTUs was represented by a connection between these vertices (edge; Appendix S2: Fig. S3). Edges are not indicative of a putative virus‐host relationship between two specific OTUs, rather the OTUs have been found in the same incubation vessel at the same time. To focus on the relationship between viruses and their prokaryotic hosts, only connections between prokaryotic and viral OTUs were allowed. We used the one‐figure parameter graph link efficiency to summarize the information within these networks. Graph link efficiency is a measure of how tightly connected a network is in relation to its number of edges, which in turn depends on the number of prokaryotic and viral OTUs. Graph link efficiency was calculated as (1 − mean graph distance)/edge count, where mean graph distance is given as the sum of the length of the shortest paths between all pairs of vertices/vertex count. Graph link efficiency is given as the average of duplicate measurements. Although network graphs may look dramatically different from each other due to differences in the number of vertices and edges, they may have a similar level of connectedness as expressed by graph link efficiency, i.e., the number of connections between prokaryotes and viruses relative to the number of prokaryotic and viral OTUs (Appendix S2: Fig. S3). Thus, given that we solely focus on connections of prokaryotic with viral OTUs, differences in graph link efficiency among samples are largely driven by differences in the number of prokaryotic relative to viral OTUs. Here, such changes could be due to the lysis of a subset of the members of the prokaryotic community producing specific types of viruses or due to enhanced growth of a specific group of prokaryotes particularly well adapted to the culture conditions. Given that different water masses harbor different communities (Galand et al. 2010, Agogué et al. 2011, Winter et al. 2013, Hurwitz et al. 2014) it is an advantage that the influence of the overall number of prokaryotic and viral OTUs is of minor importance for graph link efficiency (Appendix S2: Fig. S3). Here, the focus is solely on changes in graph link efficiency between treatments and the corresponding controls. Given that the fingerprinting methods used in this study are a crude way to characterize prokaryotic and viral communities, differences in graph link efficiency indicate that changes are pronounced enough to be detectable even by such conservative methods.

### Data treatment and statistics

Data treatment and statistics were performed within the software environment Mathematica (version 10.3; Wolfram Research). Spearman rank correlation coefficients (*r*) were calculated to test for correlations between selected parameters. For each experiment and time point and taking duplicate incubations into account, experimental data (FIC, VP, graph link efficiency) from the treatments were expressed relative to the corresponding controls (Appendix S2: Fig. S2c). Similarly, the ratio of in situ data on biological (leucine incorporations, prokaryotic and viral abundance; Appendix S2: Fig. S2d) and physical (temperature, salinity; Appendix S2: Fig. S2e) parameters was calculated between the two water masses. In the mixing control, a mixture of two prokaryotic communities was incubated in a mixture of equal volumes of ultrafiltrate from two water masses (Appendix S2: Fig. S2b). Thus, the ratios of in situ biological and physical parameters calculated from both water masses corresponding to experimental mixing treatments were reduced by half as both water masses were used in the corresponding control incubations (Appendix S2: Fig. S2d, e). Subsequently, recoded data were centered on their means column‐wise.

Redundancy and partial redundancy analyses in combination with variation partitioning was used to calculate the fraction of variation in FIC, VP, and graph link efficiency that was explained by the variation in in situ biological parameters (prokaryotic leucine incorporation, prokaryotic and viral abundance) used as explanatory variables and with water mass‐defining physical parameters (temperature, salinity) as co‐variables. Additionally, the fraction of variation in graph link efficiency explained by variations in FIC and VP as explanatory variables together with the physical parameters temperature and salinity as co‐variables was calculated. For all redundancy and partial redundancy analyses data from transplantation and mixing treatments were analyzed together. Statistical significance of the results was tested by 10,000 random permutations of residuals (Legendre and Legendre 1998). A Mann–Whitney *U* test was used to test for significant differences in specific parameters between pairs of treatments, whether changes in FIC and VP differed after 32 h and 72 h of incubation, and whether changes in bulk and cell‐specific prokaryotic leucine incorporation differed between each other in the experimental treatments. Differences based on comparing average values obtained from duplicate incubations were considered relevant if their ranges were not overlapping. Generally, results of statistical tests were assumed to be significant at *P*‐values ≤0.05.

## Results

### Water mass characteristics

Temperature and salinity in NADW varied between 2.88–3.01°C and 34.94–34.97, respectively (Table 1). Compared to NADW, MSOW and AAIW were substantially warmer and more saline, whereas AABW was colder and less saline (Appendix S2: Fig. S4). The variation of prokaryotic leucine incorporation, prokaryotic abundance, and viral abundance in NADW was consistent with a north‐south gradient (Table 1, Fig. 1; Spearman Rank correlation analyses against latitude of sampling location: for all correlations *r *≥* *0.9, *P *≤* *0.0374). Overall, prokaryotic leucine incorporation increased with prokaryotic (*r *=* *0.98, *P *≤* *0.0001) and viral abundance (*r *=* *0.81, *P *=* *0.0049). Also, prokaryotic and viral abundances were significantly positively correlated with each other (*r *=* *0.83, *P *=* *0.0029).

**Table 1:** In situ data for the sampled water masses

Station	Water mass	Depth	Temp.	Potential temp.	Salinity	Leucine	Prokaryotes	Viruses
1	NADW	2,750	2.88	2.65	34.95	230	0.3	7.3
1	MSOW	900	9.71	9.60	35.70	890	0.7	13.5
2	NADW	2,750	3.01	2.78	34.94	208	0.3	7.2
2	AAIW	1,300	6.31	6.18	35.27	922	0.6	8.9
3	NADW	2,750	2.94	2.71	34.95	145	0.2	3.5
3	AAIW	1,700	4.69	4.54	35.13	244	0.3	5.4
4	NADW	2,750	2.95	2.72	34.96	17	0.1	3.1
4	AABW	5,000	2.40	1.92	34.89	14	0.1	5.1
5	NADW	2,750	2.98	2.76	34.97	110	0.2	3.7
5	AABW	5,000	2.44	1.96	34.89	66	0.1	3.0

*Notes*

The table gives the station number (see Fig. 1), the sampled water mass (NADW, North Atlantic Deep Water; MSOW, Mediterranean Sea Overflow Water; AAIW, Antarctic Intermediate Water; AABW, Antarctic Bottom Water), sampling depth (m), temperature (temp., °C), potential temperature (potential temp., °C), salinity, prokaryotic leucine incorporation rate (×10^−15^ M/d), prokaryotic (N × 10^5^ mL^−1^) and viral abundance (N × 10^5^ mL^−1^).

### FIC and VP

FIC and VP (Appendix S2: Figs. S5a, c, e, g, i and S6a, c, e, g, i), corrected for differences between prokaryotic abundance at the onset of the experiments and in situ prokaryotic abundance, in NADW varied on average between 19–128% of prokaryotic abundance and between 1.8–23.7 × 10^3^ viruses·mL^−1^·h^−1^, respectively (Table 2). On average, FIC in MSOW was 61.5% of prokaryotic abundance, varied between 21.5–64.5% in AAIW, and ranged between 48.5–56.5% in AABW. Contrary to FIC, VP in the water masses other than NADW decreased with depth from an average of 22.5 × 10^3^ viruses·mL^−1^·h^−1^ in MSOW to 8.8–8.9 × 10^3^ viruses·mL^−1^·h^−1^ in AAIW, and to 2.9–4.2 × 10^3^ viruses·mL^−1^·h^−1^ in AABW (Table 2). After 32 h of incubation, FIC in experiments 2–3 and VP in experiment 1 were substantially higher in the mixing controls as compared to the corresponding single water mass controls (Appendix S2: Figs. S5c, e and S6a). Overall FIC after 32 h was significantly lower compared to data after 72 h of incubation (Mann–Whitney *U* test: *U *=* *7.69, *P *<* *0.0001; Appendix S2: Fig. S5), while VP did not differ significantly between the two time points (Mann–Whitney *U* test: *U *=* *0.78, *P *=* *0.4332; Appendix S2: Fig. S6).

**Table 2:** Frequency of infected cells (FIC) and viral production (VP)

Experiment/Station	Water mass	FIC			VP		
		Avg	Min	Max	Avg	Min	Max
1	NADW	128.0	113.0	143.0	23.7	20.0	27.4
1	MSOW	61.5	44.0	79.0	22.5	17.0	28.0
2	NADW	24.5	21.0	28.0	3.4	3.1	3.8
2	AAIW	21.5	18.0	25.0	8.8	7.2	10.5
3	NADW	41.5	41.0	42.0	4.1	4.0	4.1
3	AAIW	64.5	63.0	66.0	8.9	8.3	9.5
4	NADW	29.0	17.0	41.0	2.4	1.7	3.1
4	AABW	56.5	55.0	58.0	4.2	2.8	5.5
5	NADW	19.0	15.0	23.0	1.8	1.6	2.1
5	AABW	48.5	28.0	69.0	2.9	1.5	4.3

*Notes*

The table gives FIC (% of prokaryotic abundance; calculated with an assumed burst size of 30) and VP (N × 10^3^·mL^−1^·h^−1^) as estimated in the controls for the single water mass tranplantation treatments after 32 h of incubation. Data on VP have been corrected for the difference between in situ prokaryotic abundance and prokaryotic abundance at the start of the experiments (Appendix S2: Fig. S6). NADW, North Atlantic Deep Water; MSOW, Mediterranean Sea Overflow Water; AAIW, Antarctic Intermediate Water; AABW, Antarctic Bottom Water.

### Treatment effects on FIC, VP, and prokaryotic leucine incorporation

**Figure 2:**
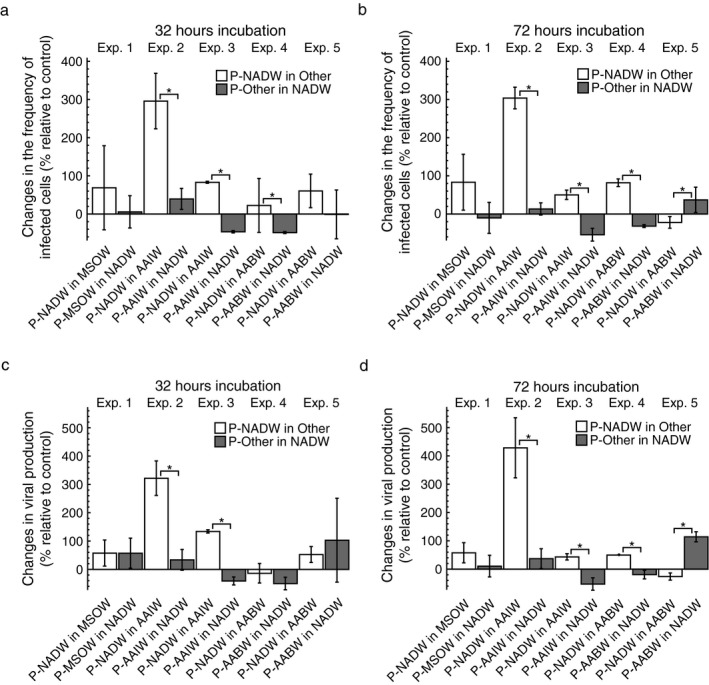
The effect of transplantation on the frequency of infected cells (FIC) and viral production (VP). The figure shows changes in FIC and VP of the transplant treatment relative to the control treatment after 32 h (a, c) and 72 h (b, d) of incubation. Prokaryotes obtained from a specific water mass are indicated by the prefix “P” followed by either North Atlantic Deep Water (NADW), Mediterranean Sea Outflow Water (MSOW), Antarctic Intermediate Water (AAIW), or Antarctic Bottom Water (AABW). Ultrafiltered water used as incubation medium in the experiments is indicated by the water masses’ abbreviation. Data are given as average values and error bars represent the standard deviation. Brackets with “*” indicate significant differences between the treatments of a specific experiment based on a Mann–Whitney *U* test.

**Figure 3:**
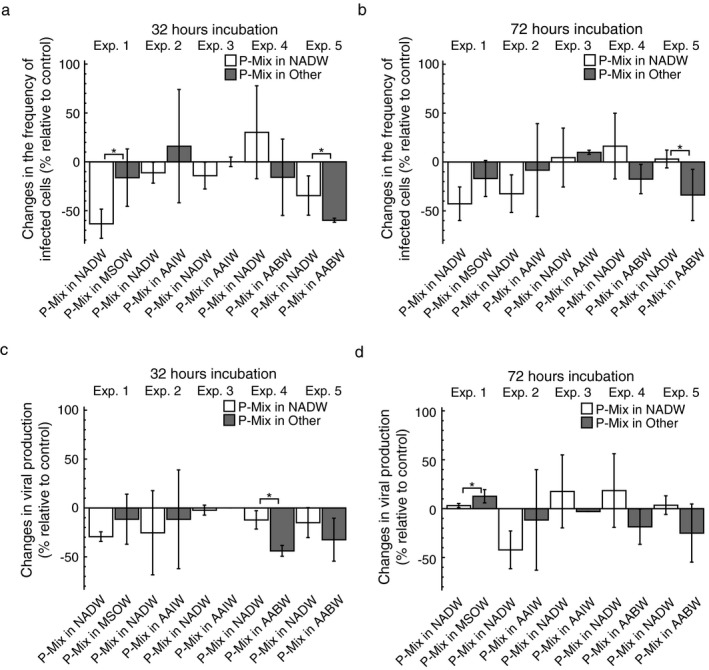
The effect of transplantation on the frequency of infected cells (FIC) and viral production (VP) in mixing treatments. The figure shows changes in FIC and VP in treatments with prokaryotes obtained from two water masses (P‐Mix) incubated in one of two water masses relative to mixing treatments incubated in a mixture of both water masses as a control after 32 h (a, c) and 72 h (b, d) of incubation. Source water masses were North Atlantic Deep Water (NADW), Mediterranean Sea Outflow Water (MSOW), Antarctic Intermediate Water (AAIW), or Antarctic Bottom Water (AABW). Data are given as average values and error bars represent the standard deviation. Brackets with “*” indicate significant differences between the treatments of a specific experiment based on a Mann–Whitney *U* test.

Changes in FIC and VP of treatments relative to controls were calculated based on estimates of FIC and VP in duplicate incubations (Figs. 2 and 3) as described above (Appendix S2: Fig. S2c). On average, FIC in transplantation treatments with prokaryotes from NADW increased by 22–296% and varied between −22 and 304% of FIC in the controls after 32 and 72 h of incubation (Fig. 2a, b). The variation of changes in FIC in treatments with prokaryotes from a single water mass in NADW relative to the controls was between −49 and 40% and between −54 and 37% after 32 and 72 h of incubation, respectively (Fig. 2a, b). Incubating prokaryotes from NADW in ultrafiltrate from any of the other water masses resulted in changes of VP relative to the controls between −13 and 322% and between −26 and 429% after 32 h and 72 h of incubation, respectively (Fig. 2c, d). Changes of VP with prokaryotes from a single water mass incubated in NADW relative to controls varied between −50 and 103% and between −52 and 114% after 32 and 72 h, respectively (Fig. 2c, d). After 32 h, changes in FIC and VP relative to controls were significantly higher when incubating prokaryotes from NADW in AAIW in experiments 2–3 as compared to the reverse transplantation treatments (Fig. 2a, c). Additionally, changes in FIC relative to controls were significantly higher when incubating prokaryotes from NADW in AABW as compared to the reverse in experiment 4 after 32 h (Fig. 2a). After 72 h of incubation, changes in FIC and VP relative to controls were positive and significantly higher when incubating prokaryotes from NADW in AAIW in experiments 2–3 and when incubating in AABW in experiment 4, yet were significantly lower and negative when incubating in AABW in experiment 5 (Fig. 2b, d).

Overall, changes in FIC and VP in mixing treatments relative to the controls were much less pronounced and often negative as compared to data obtained from single‐source transplantation treatments (Figs. 2 and 3). On average, changes of FIC in mixing treatments relative to controls varied between −63 and 30% and between −43 and 16% after 32 and 72 h of incubation, respectively (Fig. 3a, b). Similarly, changes of VP in mixing treatments relative to the controls ranged from −44 to −2% after 32 h and from −42 to 19% after 72 h of incubation (Fig. 3c, d). Consistently, changes of FIC relative to controls were significantly different between the mixing treatments of experiment 5 after 32 and 72 h of incubation (Fig. 3a, b). Additionally, changes of FIC relative to controls differed significantly between treatments in experiment 1 after 32 h (Fig. 3a) and changes of VP relative to controls significantly differed between the treatments of experiment 4 after 32 h and of experiment 1 after 72 h (Fig. 3c, d).

At the end of the incubation period, changes in prokaryotic leucine incorporation relative to controls ranged from −51 to 200% in single water mass transplantations (Appendix S2: Fig. S7a) and from −68 to 33% in mixing treatments (Appendix S2: Fig. S7c). Similarly, changes in cell‐specific prokaryotic leucine incorporation relative to controls varied between −51 and 174% and between −64 and 38% in transplantation and mixing treatments, respectively (Appendix S2: Fig. S7b, d). In experiment 3, bulk and cell‐specific prokaryotic leucine incorporation relative to the controls differed significantly between the transplantation treatments (Appendix S2: Fig. S7a, b). Overall, changes in bulk and cell‐specific prokaryotic leucine incorporation were similar in transplantation (Mann–Whitney *U* test: *U *=* *0.51, *P *=* *0.6134) and mixing treatments (Mann–Whitney *U* test: *U *=* *0.46, *P *=* *0.6476).

### Treatment effects on graph link efficiency

Changes in graph link efficiency, based on co‐occurrence networks of prokaryotic and viral fingerprints (Appendix S2: Figs. S3 and S8), were calculated relative to control treatments (Appendix S2: Fig. S2c). When incubating prokaryotes from NADW in ultrafiltered water from any of the other water masses, changes in graph link efficiency relative to the controls ranged on average between −0.23 and 0.53% after 24 h, between −0.49 and 0.51% after 48 h, and between −0.77 and 0.05% after 72 h (Appendix S2: Fig. S9a–c). On average, changes in graph link efficiency relative to controls when incubating prokaryotes from a single water mass in ultrafiltered NADW varied between −0.44 and 0.21% after 24 h, between −0.26 and 0.36% after 48 h, and between −0.18 and 0.08% after 72 h (Appendix S2: Fig. S9a–c). Throughout the entire incubation period, graph link efficiency in mixing treatments changed between −0.53 and 0.61% relative to controls (Appendix S2: Fig. S9d–f). Overall, changes in graph link efficiency did not follow a recognizable pattern as compared to changes in FIC and VP (Appendix S2: Fig. S2), and only few significant differences between treatments could be identified (Appendix S2: Fig. S9).

### Factors affecting changes in FIC, VP, and graph link efficiency

**Figure 4:**
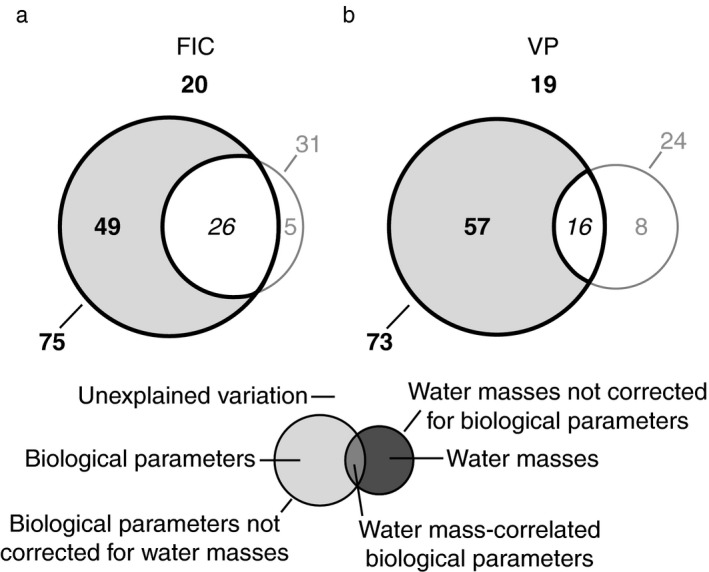
Variation partitioning of changes in the frequency of infected cells (FIC) and viral production (VP). The figure shows the percentage of the variation in FIC (a) and VP (b) relative to controls and throughout the entire incubation period (32 h and 72 h analyzed together) that is explained by in situ biological parameters (prokaryotic leucine incorporation, prokaryotic and viral abundance) corrected and uncorrected for the influence of water masses, water‐mass defining parameters (temperature, salinity) corrected and uncorrected for biological parameters, water‐mass correlated biological parameters, and that remains unexplained in the form of a Venn diagram. Data from single source transplantation and mixing treatments were analyzed together. Statistically significant values are in bold, insignificant values are in gray, and values that cannot be tested for statistical significance are in italics.

Throughout the entire incubation period, 80% of the variation in FIC and 81% of the variation in VP relative to controls were explained by models containing in situ prokaryotic leucine incorporation and prokaryotic and viral abundance as explanatory parameters in combination with temperature and salinity as water mass‐defining co‐variables (Appendix S1: Tables S1 and S2; Fig. 4). Biological parameters not corrected for the influence of water masses explained 75% of the variation in FIC and 73% of the variation in VP. Biological parameters alone accounted for 49% and 57% of the variation in FIC and VP, respectively, whereas water masses did not significantly influence either FIC or VP (Fig. 4). Similar results were obtained when analyzing data after 32 h and 72 h of incubation (Appendix S1: Tables S1 and S2).

**Figure 5:**
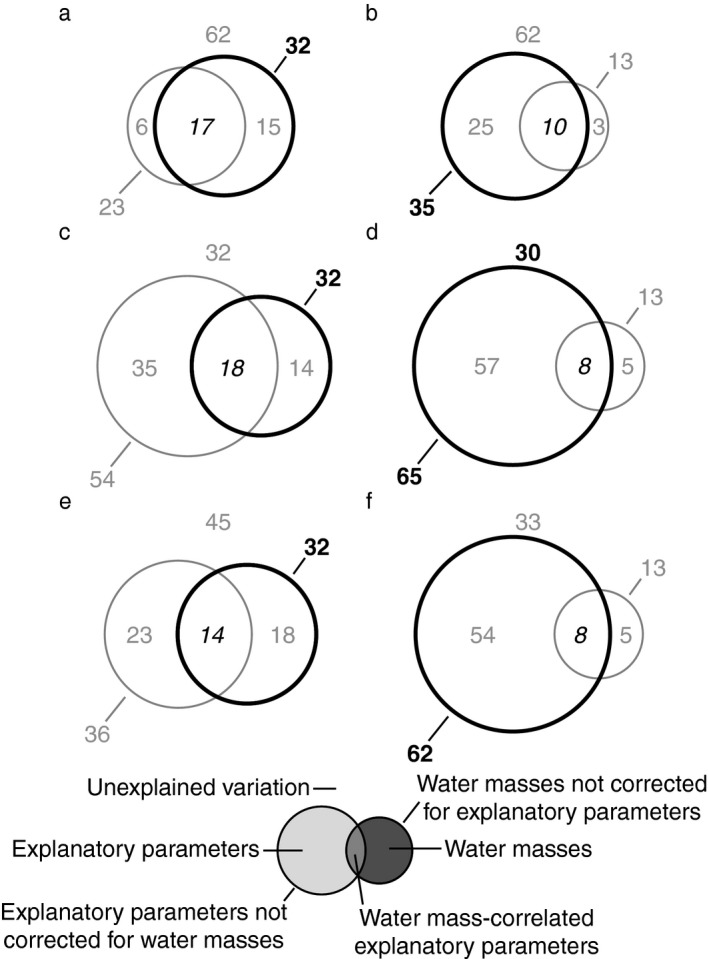
Variation partitioning of graph link efficiency. The figure shows the percentage of the variation in graph link efficiency relative to controls that is explained by water‐mass defining parameters (temperature, salinity) correct and uncorrected for explanatory parameters, explanatory parameters corrected and uncorrected for the influence of water masses, water‐mass correlated explanatory parameters, and that remains unexplained after 24 h (a, c, e) and 72 h (b, d, f) of incubation. Explanatory parameters were prokaryotic leucine incubation, prokaryotic and viral abundance (a, b), the frequency of infected cells (c, d), and viral production (e, f). Data from single source transplantation and mixing treatments were analyzed together. Statistically significant values are in bold, insignificant values are in gray, and values that cannot be tested for statistical significance are in italics.

After 24 h, the variation in graph link efficiency relative to controls was best explained by the water mass‐defining parameters temperature and salinity (32%) not corrected for the influence of either group of explanatory parameters (Fig. 5a, c, e). However, after 72 h of incubation, the variation of graph link efficiency was significantly accounted for by variation in prokaryotic leucine incorporation and prokaryotic and viral abundance not corrected for water masses (35%; Fig. 5b) and to a larger extent by FIC not corrected for water masses (65%; Fig. 5d) and VP not corrected for water masses (62%; Fig. 5f). The variation of graph link efficiency could not be explained by any of the tested models or parameters after 48 h of incubation (Appendix S1: Tables S3–S5).

## Discussion

### Consequences of the experimental set‐up for data interpretation

The experiments were designed to mimic mixing of water masses while adhering to the requirements of the virus‐dilution approach often used to estimate FIC and VP. The latter requires removing viruses from the water used as incubation medium for the prokaryotes collected by filtration to prevent new viral infections in the incubations (Murray and Jackson 1992). The viruses detected at the onset of our experiments were added as part of the prokaryotic concentrates and amounted to between 7–13% of in situ viral abundance (data not shown). On average and throughout the entire incubation period, viral abundance ranged from 9–28% and prokaryotic abundance from 27–36% of in situ abundances (data not shown). Also, equal numbers of prokaryotes and viruses were added to the treatments and the corresponding controls at the start of the incubations. Thus, in the case of new viral infections during the incubations, FIC and VP should have increased similarly in controls and treatments. However, our data clearly do not follow such a scenario (Figs. 2 and 3), suggesting that the dilution of viruses in the experiments was high enough to prevent new viral infections.

To reduce the impact of a possible volumetric bottle effect (but see also Hammes et al. 2010), the size of the incubation vessels was chosen to be as large as practically possible (20 L). Yet, the necessary filtration steps and subsequent incubations for 3 d under surface pressure conditions may have led to some artifacts. Acknowledging these pitfalls, the experiments were designed to enable correcting every treatment with its corresponding control and performing every incubation, including the controls, in duplicate (Appendix S2: Fig. S2a–c). Thus, data from duplicates afforded a handle on experimental error. Correcting all experimental data with data from their corresponding controls greatly reduced the impact of the experimental procedures on the results. Additionally, treating in situ data similarly to experimental data (Appendix S2: Figs. S2d, e and S4) enabled the statistical analyses of the effects of in situ environmental conditions on experimental results, which otherwise would not be possible (Figs. 4 and 5). Consequently, results obtained by redundancy analyses and variation partitioning in this study focus on the magnitude of changes in parameters, not on the magnitude of these parameters themselves.

For each of the five experiments, two different types of treatments were conducted: transplantation treatments and mixing treatments. Transplanting prokaryotes from one water mass into another constitutes an extreme and artificial way of mixing. The mixing treatments, where equal volumes of prokaryotes obtained from two different water masses were used as inoculum, resemble natural mixing of water masses much closer and also allow to experimentally capture potential interactions among certain members of the two different prokaryotic communities. Consequently, changes in FIC, VP, and graph link efficiency were more pronounced in transplantation compared to mixing treatments (Figs. 2 and 3; Appendix S2: Fig. S9). Nevertheless, all statistical analyses concerning the effects of biological and water mass‐defining physical parameters on FIC, VP, and graph link efficiency have been conducted with data from transplantation and mixing treatments together (Figs. 4 and 5) and the number of data points for both types of treatments was equal for all analyses (Appendix S2: Fig. S2c). Thus, significant effects detected by the analyses should be considered conservative yet come with a high degree of confidence.

### FIC and VP

#### Comparison to other aquatic environments

Overall, FIC (19.0–128.0%) and VP (1.8–23.7 × 10^3^ viruses·mL^−1^·h^−1^; Table 2) as determined in single water mass controls after 32 h of incubation were comparable to previously reported data obtained from similar depth zones in the Atlantic Ocean (De Corte et al. 2010, 2012, Fonda Umani et al. 2010, Muck et al. 2014). In comparison to data from other marine environments, VP from this study was similar to VP reported for the Canadian Arctic Shelf region (Payet and Suttle 2013) but was lower than in the Northern Baltic Sea (Holmfeldt et al. 2010), the North Sea (Winter et al. 2004, 2005), and much lower compared to the Chesapeake Bay (Winget et al. 2011).

#### FIC and the issue of burst size

The burst size, i.e., the number of progeny viruses released per lysed host cell, varies among virus types (e.g., Jiang et al. 1998, De Paepe and Taddei 2006) and consequently also among aquatic environments (Wommack and Colwell 2000, Weinbauer 2004, Parada et al. 2006) harboring different viral communities. We assumed a constant burst size of 30 to calculate FIC (burst size is not needed to calculate VP; Appendix S3: Eq. S2; Weinbauer et al. 2003, Winter et al. 2004, Parada et al. 2006). However, in some of our incubations FIC exceeded 100% of prokaryotic abundance (Table 2; Appendix S2: Fig. S5), suggesting that at least in these cases the assumed burst size of 30 was too low. While this affects absolute values of FIC, it is irrelevant for calculating changes in FIC relative to the controls, because burst size is a constant and equal factor. The overall consistent patterns between changes in FIC and VP relative to the controls confirm this notion (Figs. 2 and 3).

#### Evidence for lysogenic induction

Changes in FIC and VP relative to controls were mostly positive for transplantation treatments (Fig. 2), whereas changes in mixing treatments were mostly negative (Fig. 3). An increase in FIC and VP compared to the control means that the same prokaryotic community produced more viruses when incubated in a different water mass as compared to being incubated in the original water mass. How is this possible given that new viral infection is prevented by dilution? The switch in environmental conditions might lead to higher prokaryotic growth rates and consequently higher VP (Middelboe 2000) as compared to the controls. However, only one case was found where bulk and cell‐specific prokaryotic leucine incorporation rates as a proxy for prokaryotic growth significantly increased compared to the controls (Appendix S2: Fig. S7a, b; experiment 3). It can be argued that the method used to measure bulk prokaryotic leucine incorporation might not be sensitive enough to detect minute changes (e.g., by a small number of prokaryotic taxa), that still might cause the observed changes in FIC and VP, especially given the large variation between duplicates in some cases (Appendix S2: Fig. S7). However, even in this case and given that viral infections during the incubation period were inhibited by the dilution, FIC should remain similar to the controls as only the time needed to produce a given number of viruses would decrease causing VP to increase. This would also imply that VP should increase initially and decline again after a certain amount of time, leading to differences in the patterns of changes in FIC and VP. However, while FIC after 32 h of incubation was significantly lower than after 72 h (Appendix S2: Fig. S5), VP at both time points was similar (Appendix S2: Fig. S6). These data translate into consistent patterns of changes in FIC and VP (Figs. 2 and 3), contradicting the above hypothesis. Thus, the data are not compatible with a scenario where the experimental manipulations in some treatments led to an increase in FIC and VP compared to the controls as a response of increased prokaryotic growth. Alternatively, higher FIC and VP compared to the controls might be a consequence of induction of lysogenic viruses. Although lysogenic viruses are capable of causing death to the host cell eventually, prophages and their host cells are engaged in a mutualistic relationship (Paul 2008), where the prophages are tuned into a number of cellular sensory networks enabling them to react to a diverse set of environmental changes and stressors (Rozanov et al. 1998, Michel 2005, Shkilnyi and Koudelka 2007, Ghosh et al. 2009, Erez et al. 2017). Increases in FIC relative to the controls coincided with increases in VP (Figs. 2 and 3) as is expected when additional viruses are produced. Thus, the data support the notion that elevated FIC and VP compared to the controls were a consequence of induction of lysogenic viruses into the lytic cycle. We hypothesize that induction of prophages in our experiments was due to a RecA‐independent induction mechanism, as all incubations were performed in the dark. RecA‐independent induction mechanisms in the deep ocean might be a solution to the conundrum of large numbers of lysogens found in the deep‐sea (Weinbauer et al. 2003) in the absence of a known natural inducing agent, as ultraviolet radiation does not penetrate the deep ocean.

#### Mixing affects FIC and VP based on differences in productivity

Faster growing prokaryotes will be able to maintain higher prokaryotic and viral abundance. This notion is underlined by the finding that prokaryotic leucine incorporation rates often are positively correlated with prokaryotic and viral abundance (Wommack and Colwell 2000, this study see Results section and Table 1), allowing interpreting these parameters as a proxy for productivity. Statistical analyses revealed that changes in FIC and VP relative to the controls could largely be explained by how strongly the source water masses differed in their level of productivity (Fig. 4; Appendix S1: Tables S1 and S2). However, our data show that whether FIC and VP increase or decrease relative to the controls is not determined by whether water mass A is characterized by higher or lower levels of productivity compared to water mass B (Figs. 2 and 3; Appendix S2: Fig. S4). Additionally, it was unexpected that variations in temperature and salinity used to identify the source water masses (Appendix S2: Fig. S1) had no effect on FIC and VP (Fig. 4; Appendix S1: Tables S1 and S2), although different water masses harbor different prokaryotic and virus communities (Galand et al. 2010, Agogué et al. 2011, Winter et al. 2013, Hurwitz et al. 2014). In summary, the data indicated that pronounced differences in productivity between the source water masses related to strong changes in FIC and VP relative to the controls without determining the direction of these changes and irrespective of the composition of the affected prokaryotic communities.

### Co‐occurrence patterns of prokaryotes with viruses – graph link efficiency

#### Changes in the lytic activity of viruses affect co‐occurrence of prokaryotes with viruses

Co‐occurrence patterns of prokaryotes with viruses changed dramatically due to mixing and were initially well explained by differences in in situ temperature and salinity between the source water masses (Fig. 5a, c, e). However, changes in graph link efficiency relative to controls could not be explained by any of the measured parameters after 48 h (Appendix S1: Tables S3–S5). Yet, after 72 h changes in graph link efficiency were explained well by differences in productivity‐related parameters (prokaryotic leucine incorporation, prokaryotic and viral abundance) between source water masses (Fig. 5b), but above all, by changes in FIC and VP (Fig. 5d, f). Given that specific OTUs will only become detectable when their relative abundance rises above a certain threshold level or disappear when their relative abundance falls below the detection limit, our data indicate that changes in co‐occurrence patterns of prokaryotes with viruses need more time to manifest themselves in the fingerprints (Appendix S1: Tables S1 and S2). Thus, our data agree with the notion that different water masses harbor different prokaryotic and viral communities (Galand et al. 2010, Agogué et al. 2011, Winter et al. 2013, Hurwitz et al. 2014), as such differences encoded in the co‐occurrence patterns of prokaryotes with viruses were still detectable after 24 h of incubation (Fig. 5a, c, e). An intermediate state was reached after 48 h of incubation, where changes in co‐occurrence patterns of prokaryotes with viruses relative to controls were no longer attributable to differences in salinity and temperature and where the effect of mixing was not yet acting long enough to have a clear effect on graph link efficiency (Appendix S1: Tables S3–S5). Eventually, more than 60% of the variability in changes in graph link efficiency relative to controls was explained by changes in FIC and VP (Fig. 5d, f) and only 35% by differences in productivity‐related parameters between the source water masses (Fig. 5b). These data suggest that upon mixing, differences in productivity‐related parameters between the source water masses determine changes in FIC and VP (Fig. 4) which, in turn, have a strong influence on changes in co‐occurrence patterns of prokaryotes with viruses by altering the relative abundance of specific prokaryotic and viral OTUs due to changes in the lytic activity of specific viruses.

## Conclusions

Our initial hypothesis was that mixing of deep ocean water masses enhances the lytic activity of viruses infecting prokaryotes due to enhanced growth of some members of the prokaryotic community that are well adapted to the altered environmental conditions, allowing them to produce viruses faster. Although, in most cases mixing increased FIC and VP there was no evidence that differences in prokaryotic growth rates caused these changes. Instead, strong differences between the source water masses in productivity‐related parameters (prokaryotic leucine incorporation, prokaryotic and viral abundance) resulted in strong changes in FIC and VP. Surprisingly, changes in FIC and VP were largely independent of the identity of the source water masses and, thus, of the composition of prokaryotic and viral communities. Given that changes in FIC and VP were consistent with each other, enhanced lytic activity of viruses as a consequence of mixing was caused by the induction of lysogenic viruses. This finding may explain the large numbers of lysogenized prokaryotic cells in the deep ocean, an environment devoid of the strong natural inducing agent ultraviolet radiation (Weinbauer et al. 2003, Paul 2008). Upon mixing of water masses, changes in the lytic activity of viruses altered the relative abundance of specific prokaryotic and viral taxa as indicated by the changes in co‐occurrence patterns of prokaryotes with viruses. In conclusion, mixing of deep ocean water masses is disrupting the delicate balance between viruses and their prokaryotic host cells. Often, mixing in the deep ocean causes the induction of lysogenic viruses, reducing the efficiency of the microbial loop, by which DOC is transfered to higher trophic levels.

## References

[ecy2135-bib-0001] Agogué, H. , D. Lamy , P. R. Neal , M. Sogin , and G. J. Herndl . 2011 Water mass‐specificity of bacterial communities in the North Atlantic revealed by massively parallel sequencing. Molecular Ecology 20:258–274. 21143328 10.1111/j.1365-294X.2010.04932.x PMC3057482

[ecy2135-bib-0002] Azam, F. , T. Fenchel , J. G. Field , J. S. Gray , L. A. Meyer‐Reil , and F. Thingstad . 1983 The ecological role of water‐column microbes in the sea. Marine Ecology Progress Series 10:257–263.

[ecy2135-bib-0003] Béthoux, J. P. , B. Gentili , J. Raunet , and D. Tailliez . 1990 Warming trend in the western Mediterranen deep water. Nature 347:660–662.

[ecy2135-bib-0004] Bettarel, Y. , et al. 2011 Viral distribution and life strategies in the Bach Dang Estuary, Vietnam. Microbial Ecology 62:143–154. 21390531 10.1007/s00248-011-9835-6

[ecy2135-bib-0005] Brussaard, C. P. D. , J. P. Payet , C. Winter , and M. G. Weinbauer . 2010 Quantification of aquatic viruses by flow cytometry Pages 102–109 *in* Wilhelm S. W., Weinbauer M. G., and Suttle C. A., editors. Manual of aquatic viral ecology. ASLO, Waco, Texas, USA.

[ecy2135-bib-0006] Bryden, H. L. , and A. J. G. Nurser . 2003 Effects of strait mixing on ocean stratification. Journal of Physical Oceanography 33:1870–1872.

[ecy2135-bib-0007] Chen, Y. , I. Golding , S. Sawai , L. Guo , and E. C. Cox . 2005 Population fitness and the regulation of *Escherichia coli* genes by bacterial viruses. PLoS Biology 3:1276–1282. 10.1371/journal.pbio.0030229 PMC1151598 15984911

[ecy2135-bib-0008] Cissoko, M. , A. Desnues , M. Bouvy , T. Sime‐Ngando , E. Verling , and Y. Bettarel . 2008 Effects of freshwater and seawater mixing on virio‐ and bacterioplankton in a tropical estuary. Freshwater Biology 53:1154–1162.

[ecy2135-bib-0009] De Corte, D. , E. Sintes , C. Winter , T. Yokokawa , T. Reinthaler , and G. J. Herndl . 2010 Links between viral and prokaryotic communities throughout the water column in the (sub)tropical Atlantic Ocean. ISME Journal 4:1431–1442. 20485386 10.1038/ismej.2010.65

[ecy2135-bib-0010] De Corte, D. , E. Sintes , T. Yokokawa , T. Reinthaler , and G. J. Herndl . 2012 Links between viruses and prokaryotes throughout the water column along a North Atlantic latitudinal transect. ISME Journal 6:1566–1577. 22258100 10.1038/ismej.2011.214 PMC3400414

[ecy2135-bib-0011] De Paepe, M. , and F. Taddei . 2006 Viruses’ life history: towards a mechanistic basis of a trade‐off between survival and reproduction among phages. PLoS Biology 4:e193. 16756387 10.1371/journal.pbio.0040193 PMC1475768

[ecy2135-bib-0012] DeLong, E. F. 1992 Archaea in coastal marine environments. Proceedings of the National Academy of Sciences USA 89:5685–5689. 10.1073/pnas.89.12.5685 PMC49357 1608980

[ecy2135-bib-0013] Emery, W. J. 2001 Water types and water masses Pages 3179–3187 *in* Steele J. H., Thorpe S. A., and Turekian K. K., editors. Encyclopedia of ocean sciences. Academic Press, San Diego, California, USA.

[ecy2135-bib-0014] Erez, Z. , et al. 2017 Communication between viruses guides lysis‐lysogeny decisions. Nature 541:488–493. 28099413 10.1038/nature21049 PMC5378303

[ecy2135-bib-0015] Ferron, B. , H. Mercier , and K. Speer . 1998 Mixing in the Romanche fracture zone. Journal of Physical Oceanography 28:1929–1945.

[ecy2135-bib-0016] Finke, J. F. , B. P. V. Hunt , C. Winter , E. C. Carmack , and C. A. Suttle . 2017 Nutrients and other environmental factors influence virus abundance across oxic and hypoxic marine environments. Viruses 9:152. 10.3390/v9060152 PMC5490827 28629143

[ecy2135-bib-0017] Fonda Umani, S. , E. Malisana , F. Focaracci , M. Magagnini , C. Corinaldesi , and R. Danovaro . 2010 Disentangling the effect of viruses and nanoflagellates on prokaryotes in bathypelagic waters of the Mediterranean Sea. Marine Ecology Progress Series 418:73–85.

[ecy2135-bib-0018] Fuhrman, J. A. 1999 Marine viruses and their biogeochemical and ecological effects. Nature 399:541–548. 10376593 10.1038/21119

[ecy2135-bib-0019] Fuhrman, J. A. , I. Hewson , M. S. Schwalbach , J. A. Steele , M. V. Brown , and S. Naeem . 2006 Annually reoccurring bacterial communities are predictable from ocean conditions. Proceedings of the National Academy of Sciences USA 103:13104–13109. 10.1073/pnas.0602399103 PMC1559760 16938845

[ecy2135-bib-0020] Galand, P. E. , M. Potvin , E. O. Casamayor , and C. Lovejoy . 2010 Hydrography shapes bacterial biogeography of the deep Arctic Ocean. ISME Journal 4:564–576. 20010630 10.1038/ismej.2009.134

[ecy2135-bib-0021] Ghosh, D. , K. Roy , K. E. Williamson , S. Srinivasiah , K. E. Wommack , and M. Radosevich . 2009 Acyl‐homoserine lactones can induce virus production in lysogenic bacteria: an alternative paradigm for prophage induction. Applied and Environmental Microbiology 75:7142–7152. 19783745 10.1128/AEM.00950-09 PMC2786502

[ecy2135-bib-0022] Hammes, F. , M. Vital , and T. Egli . 2010 Critical evaluation of the volumetric “bottle effect” on microbial batch growth. Applied Environmental Microbiology 76:1278–1281. 20023110 10.1128/AEM.01914-09 PMC2820953

[ecy2135-bib-0023] Hewson, I. , and J. A. Fuhrman . 2007 Covariation of viral parameters with bacterial assemblage richness and diversity in the water column and sediments. Deep‐Sea Research I 54:811–830.

[ecy2135-bib-0024] Holmfeldt, K. , M. Middelboe , O. Nybroe , and L. Riemann . 2007 Large variabilities in host strain susceptibility and phage host range govern interactions between lytic marine phages and their Flavobacterium hosts. Applied Environmental Microbiology 73:6730–6739. 17766444 10.1128/AEM.01399-07 PMC2074958

[ecy2135-bib-0025] Holmfeldt, K. , J. Titelman , and L. Riemann . 2010 Virus production and lysate recycling in different sub‐basins of the Northern Baltic Sea. Microbial Ecology 60:572–580. 20407893 10.1007/s00248-010-9668-8

[ecy2135-bib-0026] Hurwitz, B. L. , A. H. Westveld , J. R. Brum , and M. B. Sullivan . 2014 Modeling ecological drivers in marine viral communities using comparative metagenomics and network analyses. Proceedings of the National Academy of Sciences USA 111:10714–10719. 10.1073/pnas.1319778111 PMC4115555 25002514

[ecy2135-bib-0027] Janse, I. , J. Bok , and G. Zwart . 2004 A simple remedy against artificial double bands in denaturing gradient gel electrophoresis. Journal of Microbiological Methods 57:279–281. 15063068 10.1016/j.mimet.2003.12.006

[ecy2135-bib-0028] Jiang, S. C. , and J. H. Paul . 1996 Occurence of lysogenic bacteria in marine microbial communities as determined by prophage induction. Marine Ecology Progress Series 142:27–38.

[ecy2135-bib-0029] Jiang, S. C. , C. A. Kellogg , and J. H. Paul . 1998 Characterization of marine temperate phage‐host systems isolated from Mamala Bay, Oahu, Hawaii. Applied and Environmental Microbiology 64:535–542. 9464390 10.1128/aem.64.2.535-542.1998 PMC106079

[ecy2135-bib-0030] Jover, L. F. , T. C. Effler , A. Buchan , S. W. Wilhelm , and J. S. Weitz . 2014 The elemental composition of virus particles: implications for marine biogeochemical cycles. Nature Reviews Microbiology 12:519–528. 10.1038/nrmicro3289 24931044

[ecy2135-bib-0031] Köstner, N. , L. Scharnreitner , K. Jürgens , M. Labrenz , G. J. Herndl , and C. Winter . 2017 High viral abundance as a consequence of low viral decay in the Baltic Sea redoxcline. PLoS ONE 12:e0178467. 28594863 10.1371/journal.pone.0178467 PMC5464540

[ecy2135-bib-0032] Lane, D. J. 1991 16S/23S rRNA sequencing Pages 115–176 *in* Stackebrandt E. and Goodfellow M., editors. Nucleic acid techniques in bacterial systematics. John Wiley & Sons Inc, New York, New York, USA.

[ecy2135-bib-0033] Larsen, A. , G. A. Fonnes Flaten , R.‐A. Sandaa , T. Castberg , R. Thyrhaug , S. R. Erga , S. Jacquet , and G. Bratbak . 2004 Spring phytoplankton bloom dynamics in Norwegian coastal waters: microbial community succession and diversity. Limnology and Oceanography 49:180–190.

[ecy2135-bib-0034] Legendre, P. , and L. Legendre . 1998 Numerical ecology. Elsevier, Amsterdam, The Netherlands.

[ecy2135-bib-0035] Lozovatsky, I. D. , H. J. S. Fernando , and S. M. Shapovalov . 2008 Deep‐ocean mixing on the basin scale: inference from North Atlantic transects. Deep‐Sea Research I 55:1075–1089.

[ecy2135-bib-0036] MacKinnon, J. A. , T. M. S. Johnston , and R. Pinkel . 2008 Strong transport and mixing of deep water through the Southwest Indian Ridge. Nature Geoscience 1:755–758.

[ecy2135-bib-0037] Magagnini, M. , C. Corinaldesi , L. S. Monticelli , E. DeDomenico , and R. Danovaro . 2007 Viral abundance and distribution in mesopelagic and bathypelagic waters of the Mediterranean Sea. Deep‐Sea Research I 54:1209–1220.

[ecy2135-bib-0038] Marie, D. , F. Partensky , D. Vaulot , and C. P. D. Brussaard . 1999 Enumeration of phytoplankton, bacteria, and viruses in marine samples Pages 11.11.1–11.11.15 *in* Robinson J. P., Darzynkiewicz Z., Dean P. N., Orfao A., Rabinovitch P. S., Stewart C. C., Tanke H. J., and Wheeless L. L., editors. Current protocols in cytometry. John Wiley & Sons Inc, New York, New York, USA. 10.1002/0471142956.cy1111s10 18770685

[ecy2135-bib-0039] Mei, M. L. , and R. Danovaro . 2004 Virus production and life strategies in aquatic sediments. Limnology and Oceanography 49:459–470.

[ecy2135-bib-0040] Michel, B. 2005 After 30 years of study, the bacterial SOS response still surprises us. PLoS Biology 3:1174–1176. 10.1371/journal.pbio.0030255 PMC1174825 16000023

[ecy2135-bib-0041] Middelboe, M. 2000 Bacterial growth rate and marine virus‐host dynamics. Microbial Ecology 40:114–124. 11029080 10.1007/s002480000050

[ecy2135-bib-0042] Muck, S. , T. Griessler , N. Köstner , A. Klimiuk , C. Winter , and G. J. Herndl . 2014 Fracture zones in the Mid Atlantic Ridge lead to alterations in prokaryotic and viral parameters in deep‐water masses. Frontiers in Microbiology 5:264. 24917857 10.3389/fmicb.2014.00264 PMC4040922

[ecy2135-bib-0043] Murray, A. G. , and G. A. Jackson . 1992 Viral dynamics: a model of the effects of size, shape, motion and abundance of single‐celled planktonic organisms and other particles. Marine Ecology Progress Series 89:103–116.

[ecy2135-bib-0044] Nanda, A. M. , K. Thormann , and J. Frunzke . 2015 Impact of spontaneous prophage induction on the fitness of bacterial populations and host‐microbe interactions. Journal of Bacteriology 197:410–419. 25404701 10.1128/JB.02230-14 PMC4285972

[ecy2135-bib-0045] Neilan, B. A. 1995 Identification and phylogenetic analysis of toxigenic cyanobacteria by multiplex randomly amplified polymorphic DNA PCR. Applied and Environmental Microbiology 61:2286–2291. 16535049 10.1128/aem.61.6.2286-2291.1995 PMC1388467

[ecy2135-bib-0046] Ortmann, A. C. , and C. A. Suttle . 2005 High abundances of viruses in a deep‐sea hydrothermal vent system indicates viral mediated microbial mortality. Deep‐Sea Research I 52:1515–1527.

[ecy2135-bib-0047] Parada, V. , G. J. Herndl , and M. G. Weinbauer . 2006 Viral burst size of heterotrophic prokaryotes in aquatic systems. Journal of the Marine Biological Association of the United Kingdom 86:613–621.

[ecy2135-bib-0048] Paul, J. H. 2008 Prophages in marine bacteria: dangerous molecular time bombs or the key to survival in the seas? ISME Journal 2:579–589. 18521076 10.1038/ismej.2008.35

[ecy2135-bib-0049] Payet, J. P. , and C. A. Suttle . 2013 To kill or not to kill: the balance between lytic and lysogenic viral infection is driven by trophic status. Limnology and Oceanography 58:465–474.

[ecy2135-bib-0050] Polzin, K. L. , K. G. Speer , J. M. Toole , and R. W. Schmitt . 1996 Intense mixing of Antarctic Bottom Water in the equatorial Atlantic Ocean. Nature 380:54–57.

[ecy2135-bib-0051] Pomeroy, L. R. 1974 The ocean's food web, a changing paradigm. BioScience 24:499–504.

[ecy2135-bib-0052] Reinthaler, T. , H. M. van Aken , and G. J. Herndl . 2010 Major contribution of autotrophy to microbial carbon cycling in the deep North Atlantic's interior. Deep Sea Research Part II: Topical Studies in Oceanography 57:1572–1580.

[ecy2135-bib-0053] Rodriguez‐Valera, F. , A.‐B. Martin‐Cuadrado , B. Rodriguez‐Brito , L. Pašić , T. F. Thingstad , F. Rohwer , and A. Mira . 2009 Explaining microbial population genomics through phage predation. Nature Reviews Microbiology 7:828–836. 19834481 10.1038/nrmicro2235

[ecy2135-bib-0054] Rozanov, D. V. , R. D'Ari , and S. P. Sineoky . 1998 RecA‐independent pathways of lambdoid prophage induction in *Escherichia coli* . Journal of Bacteriology 180:6306–6315. 9829941 10.1128/jb.180.23.6306-6315.1998 PMC107717

[ecy2135-bib-0055] Shkilnyi, P. , and G. B. Koudelka . 2007 Effect of salt shock on stability of lambdaimm434 lysogens. Journal of Bacteriology 189:3115–3123. 17307857 10.1128/JB.01857-06 PMC1855845

[ecy2135-bib-0056] St Laurent, L. C. , and A. M. Thurnherr . 2007 Intense mixing of lower thermocline water on the crest of the Mid‐Atlantic Ridge. Nature 448:680–683. 17687321 10.1038/nature06043

[ecy2135-bib-0057] Sullivan, M. B. , J. B. Waterbury , and S. W. Chisholm . 2003 Cyanophages infecting the oceanic cyanobacterium Prochlorococcus. Nature 424:1047–1051. 12944965 10.1038/nature01929

[ecy2135-bib-0059] Suttle, C. A. 2007 Marine viruses – major players in the global ecosystem. Nature Reviews Microbiology 5:801–812. 17853907 10.1038/nrmicro1750

[ecy2135-bib-0060] Thingstad, T. F. 2000 Elements of a theory for the mechanisms controlling abundance, diversity, and biogeochemical role of lytic bacterial viruses in aquatic systems. Limnology and Oceanography 45:1320–1328.

[ecy2135-bib-0061] van Aken, H. M. 2000a The hydrography of the mid‐latitude northeast Atlantic Ocean I: the deep water masses. Deep‐Sea Research I 47:757–788.

[ecy2135-bib-0062] van Aken, H. M. 2000b The hydrography of the mid‐latitude Northeast Atlantic Ocean II: the intermediate water masses. Deep‐Sea Research I 47:789–824.

[ecy2135-bib-0063] Weinbauer, M. G. 2004 Ecology of prokaryotic viruses. FEMS Microbiology Reviews 28:127–181. 15109783 10.1016/j.femsre.2003.08.001

[ecy2135-bib-0064] Weinbauer, M. G. , and C. A. Suttle . 1999 Lysogeny and prophage induction in coastal and offshore bacterial communities. Aquatic Microbial Ecology 18:217–225.

[ecy2135-bib-0065] Weinbauer, M. G. , I. Brettar , and M. G. Höfle . 2003 Lysogeny and virus‐induced mortality of bacterioplankton in surface, deep, and anoxic marine waters. Limnology and Oceanography 48:1457–1465.

[ecy2135-bib-0066] Wilhelm, S. W. , and C. A. Suttle . 1999 Viruses and nutrient cycles in the sea. BioScience 49:781–788.

[ecy2135-bib-0067] Williamson, S. J. , and J. H. Paul . 2004 Nutrient stimulation of lytic phage production in bacterial populations of the Gulf of Mexico. Aquatic Microbial Ecology 36:9–17.

[ecy2135-bib-0068] Williamson, S. J. , and J. H. Paul . 2006 Environmental factors that influence the transition from lysogenic to lytic existence in the phiHSIC/Listonella pelagia marine phage‐host system. Microbial Ecology 52:217–225. 16897298 10.1007/s00248-006-9113-1

[ecy2135-bib-0069] Winget, D. M. , R. R. Helton , K. E. Williamson , S. R. Bench , S. J. Williamson , and K. E. Wommack . 2011 Repeating patterns of virioplankton production within an estuarine ecosystem. Proceedings of the National Academy of Sciences USA 108:11506–11511. 10.1073/pnas.1101907108 PMC3136265 21709214

[ecy2135-bib-0070] Winter, C. , and M. G. Weinbauer . 2010 Randomly amplified polymorphic DNA reveals tight links between viruses and microbes in the bathypelagic zone of the North‐ western Mediterranean Sea. Applied and Environmental Microbiology 76:6724–6732. 20729320 10.1128/AEM.00531-10 PMC2953038

[ecy2135-bib-0071] Winter, C. , G. J. Herndl , and M. G. Weinbauer . 2004 Diel cycles in viral infection of bacterioplankton in the North Sea. Aquatic Microbial Ecology 35:207–216.

[ecy2135-bib-0072] Winter, C. , A. Smit , T. Szoeke‐Dénes , G. J. Herndl , and M. G. Weinbauer . 2005 Modelling viral impact on bacterioplankton in the North Sea using artificial neural networks. Environmental Microbiology 7:881–893. 15892707 10.1111/j.1462-2920.2005.00768.x

[ecy2135-bib-0073] Winter, C. , M.‐E. Kerros , and M. G. Weinbauer . 2009a Seasonal and depth‐related dynamics of prokaryotes and viruses in surface and deep waters of the northwestern Mediterranean Sea. Deep‐Sea Research I 56:1972–1982.

[ecy2135-bib-0074] Winter, C. , M.‐E. Kerros , and M. G. Weinbauer . 2009b Seasonal changes of bacterial and archaeal communities in the dark ocean: Evidence from the Mediterranean Sea. Limnology and Oceanography 54:160–170.

[ecy2135-bib-0075] Winter, C. , T. Bouvier , M. G. Weinbauer , and T. F. Thingstad . 2010 Trade‐offs between competition and defense specialists among unicellular planktonic organisms: the “Killing the Winner” hypothesis revisited. Microbiology and Molecular Biology Reviews 74:42–57. 20197498 10.1128/MMBR.00034-09 PMC2832346

[ecy2135-bib-0076] Winter, C. , B. Matthews , and C. A. Suttle . 2013 Effects of environmental variation and spatial distance on Bacteria, Archaea, and viruses in sub‐polar and arctic waters. ISME Journal 7:1507–1518. 23552622 10.1038/ismej.2013.56 PMC3721122

[ecy2135-bib-0077] Wommack, K. E. , and R. R. Colwell . 2000 Virioplankton: viruses in aquatic ecosystems. Microbiology and Molecular Biology Reviews 64:69–114. 10704475 10.1128/mmbr.64.1.69-114.2000 PMC98987

