# Supplementary material for: Mixing alters the lytic activity of viruses in the dark ocean
Source: Ecology. 2018 Feb 6;99(3):700–13. doi: 10.1002/ecy.2135 (PMC5905300; doi:10.1002/ecy.2135)
Supplement: Supplementary file 1 [file ECY-99-700-s001.pdf]

Mixing alters the lytic activity of viruses in the dark ocean

Christian Winter, Nicole Köstner, Carl-Philip Kruspe, Damaris Urban, Simone Muck, Thomas Reinthaler, Gerhard J. Herndl

Appendix S1

Table S1: Variation partitioning of the frequency of infected cells (FIC)

Table S2: Variation partitioning of viral production (VP)

Table S3: Variation partitioning of differences in co-occurrence patterns of prokaryotes with viruses explained by *in situ* biological and water mass-defining parameters

Table S4: Variation partitioning of differences in co-occurrence patterns of prokaryotes with viruses explained by the frequency of infected cells and water mass-defining parameters as co-variables

Table S5: Variation partitioning of differences in co-occurrence patterns of prokaryotes with viruses explained by viral production and water mass-defining parameters as co-variables

Table S1: Variation partitioning of the frequency of infected cells (FIC). The table gives the percentage of the variation in FIC relative to controls that is explained by *in situ* biological and water mass-defining parameters. Data for single source transplantation and mixing treatments were analyzed together. The matrix of biological parameters consisted of prokaryotic leucine incorporation, prokaryotic and viral abundance. Temperature and salinity were used as co-variables representing water masses. Data are presented after 32 h and 72 h of incubation and throughout the entire incubation period. The results are assumed to be significant at $p \leq 0.05$ (n.a.: not applicable).

Parameters	32 h		72 h		All	
	Fraction	*p*	Fraction	*p*	Fraction	*p*
Biological parameters and water masses	75	<0.0001	86	<0.0001	80	<0.0001
Biological parameters not corrected for water masses	68	<0.0001	82	<0.0001	75	<0.0001
Water masses not corrected for biological parameters	28	0.0925	33	0.0923	31	0.0940
Biological parameters	46	0.0072	53	0.0014	49	0.0038
Water mass-correlated biological parameters	22	n.a.	29	n.a.	26	n.a.
Water masses	6	0.2008	4	0.1881	5	0.1957
Unexplained	25	n.a.	14	n.a.	20	n.a.

Table S2: Variation partitioning of viral production (VP). The table gives the percentage of the variation in VP relative to controls that is explained by *in situ* biological and water mass-defining parameters. Data for single source transplantation and mixing treatments were analyzed together. The matrix of biological parameters consisted of prokaryotic leucine incorporation, prokaryotic and viral abundance. Temperature and salinity were used as co-variables representing water masses. Data are presented after 32 h and 72 h of incubation and throughout the entire incubation period. The results are assumed to be significant at $p \leq 0.05$ (n.a.: not applicable).

Parameters	32 h		72 h		All	
	Fraction	*p*	Fraction	*p*	Fraction	*p*
Biological parameters and water masses	70	0.0002	90	0.0001	81	<0.0001
Biological parameters not corrected for water masses	60	0.0027	85	0.0001	73	<0.0001
Water masses not corrected for biological parameters	26	0.1009	23	0.1166	24	0.1190
Biological parameters	44	0.0504	67	0.0007	57	0.0054
Water mass-correlated biological parameters	16	n.a.	18	n.a.	16	n.a.
Water masses	10	0.1649	5	0.1421	8	0.1615
Unexplained	30	n.a.	10	n.a.	19	n.a.

Table S3: Variation partitioning of differences in co-occurrence patterns of prokaryotes with viruses. The table gives the percentage of the variation in graph link efficiency of networks based on co-occurrence patterns of prokaryotes with viruses relative to controls that is explained by *in situ* biological and water mass-defining parameters. Data for single source transplantation and mixing treatments were analyzed together. The matrix of biological parameters consisted of prokaryotic leucine incorporation, prokaryotic and viral abundance. Temperature and salinity were used as co-variables representing water masses. Data are presented after 24 h, 48 h, 72 h of incubation, and throughout the entire incubation period. The results are assumed to be significant at $p \leq 0.05$ (n.a.: not applicable).

Parameters	24 h		48 h		72 h		All	
	Fraction	*p*	Fraction	*p*	Fraction	*p*	Fraction	*p*
Biological parameters and water masses	38	0.2101	24	0.5463	38	0.1473	34	0.2918
Biological parameters not corrected for water masses	23	0.2321	12	0.6291	35	0.0297	21	0.2812
Water masses not corrected for biological parameters	32	0.0222	7	0.6531	13	0.2997	18	0.1362
Biological parameters	6	0.7721	17	0.4030	25	0.1889	16	0.4271
Water mass-correlated biological parameters	17	n.a.	-5	n.a.	10	n.a.	5	n.a.
Water masses	15	0.2597	12	0.3721	3	0.5606	13	0.3287
Unexplained	62	n.a.	76	n.a.	62	n.a.	66	n.a.

Table S4: Variation partitioning of differences in co-occurrence patterns of prokaryotes with viruses. The table gives the percentage of the variation in graph link efficiency of networks based on co-occurrence patterns of prokaryotes with viruses relative to controls that is explained by the variation in the frequency of infected cells (FIC) throughout the entire incubation period as explanatory variable and temperature and salinity as co-variables representing water masses. Data for single source transplantation and mixing treatments were analyzed together. Data are presented after 24 h, 48 h, 72 h of incubation, and throughout the entire incubation period. The results are assumed to be significant at $p \leq 0.05$ (n.a.: not applicable).

Parameters	24 h		48 h		72 h		All	
	Fraction	*p*	Fraction	*p*	Fraction	*p*	Fraction	*p*
FIC and water masses	68	0.2418	60	0.2334	70	0.0373	69	0.1071
FIC not corrected for water masses	54	0.2651	50	0.2122	65	0.0091	58	0.0875
Water masses not corrected for FIC	32	0.0201	7	0.6481	13	0.3019	17	0.1356
FIC	35	0.5118	53	0.1973	57	0.0648	52	0.1049
FIC correlated with water masses	18	n.a.	-3	n.a.	8	n.a.	6	n.a.
Water masses	14	0.2175	10	0.3662	5	0.5476	11	0.2410
Unexplained	32	n.a.	40	n.a.	30	n.a.	31	n.a.

Table S5: Variation partitioning of differences in co-occurrence patterns of prokaryotes with viruses. The table gives the percentage of the variation in graph link efficiency of networks based on co-occurrence patterns of prokaryotes with viruses relative to controls that is explained by the variation in viral production (VP) throughout the entire incubation period as explanatory variable and temperature and salinity as co-variables representing water masses. Data for single source transplantation and mixing treatments were analyzed together. Data are presented after 24 h, 48 h, 72 h of incubation, and throughout the entire incubation period. The results are assumed to be significant at $p \leq 0.05$ (n.a.: not applicable).

Parameters	24 h		48 h		72 h		All	
	Fraction	*p*	Fraction	*p*	Fraction	*p*	Fraction	*p*
VP and water masses	55	0.6029	57	0.3280	67	0.0815	60	0.4105
VP not corrected for water masses	36	0.7974	51	0.1739	62	0.0199	50	0.3385
Water masses not corrected for VP	32	0.0242	7	0.6677	13	0.3025	18	0.1417
VP	23	0.9331	50	0.2726	54	0.1392	42	0.5191
VP correlated with water masses	14	n.a.	1	n.a.	8	n.a.	8	n.a.
Water masses	18	0.2495	6	0.6479	5	0.6246	10	0.5451
Unexplained	45	n.a.	43	n.a.	33	n.a.	40	n.a.
