# Supplementary material for: Mixing alters the lytic activity of viruses in the dark ocean
Source: Ecology. 2018 Feb 6;99(3):700–13. doi: 10.1002/ecy.2135 (PMC5905300; doi:10.1002/ecy.2135)
Supplement: Supplementary file 2 [file ECY-99-700-s002.pdf]

Mixing alters the lytic activity of viruses in the dark ocean

Christian Winter, Nicole Köstner, Carl-Philip Kruspe, Damaris Urban, Simone Muck, Thomas Reinthaler, Gerhard J. Herndl

Appendix S2

Figure S1: Vertical temperature-salinity profiles

Figure S2: Experimental design and data treatment

Figure S3: Examples of co-occurrence networks

Figure S4: Differences in physico-chemical and biological parameters between water masses

Figure S5: Frequency of infected cells (FIC)

Figure S6: Viral production (VP)

Figure S7: The effect of transplantation on prokaryotic leucine incorporation

Figure S8: Graph link efficiency

Figure S9: Effects of transplantation on graph link efficiency

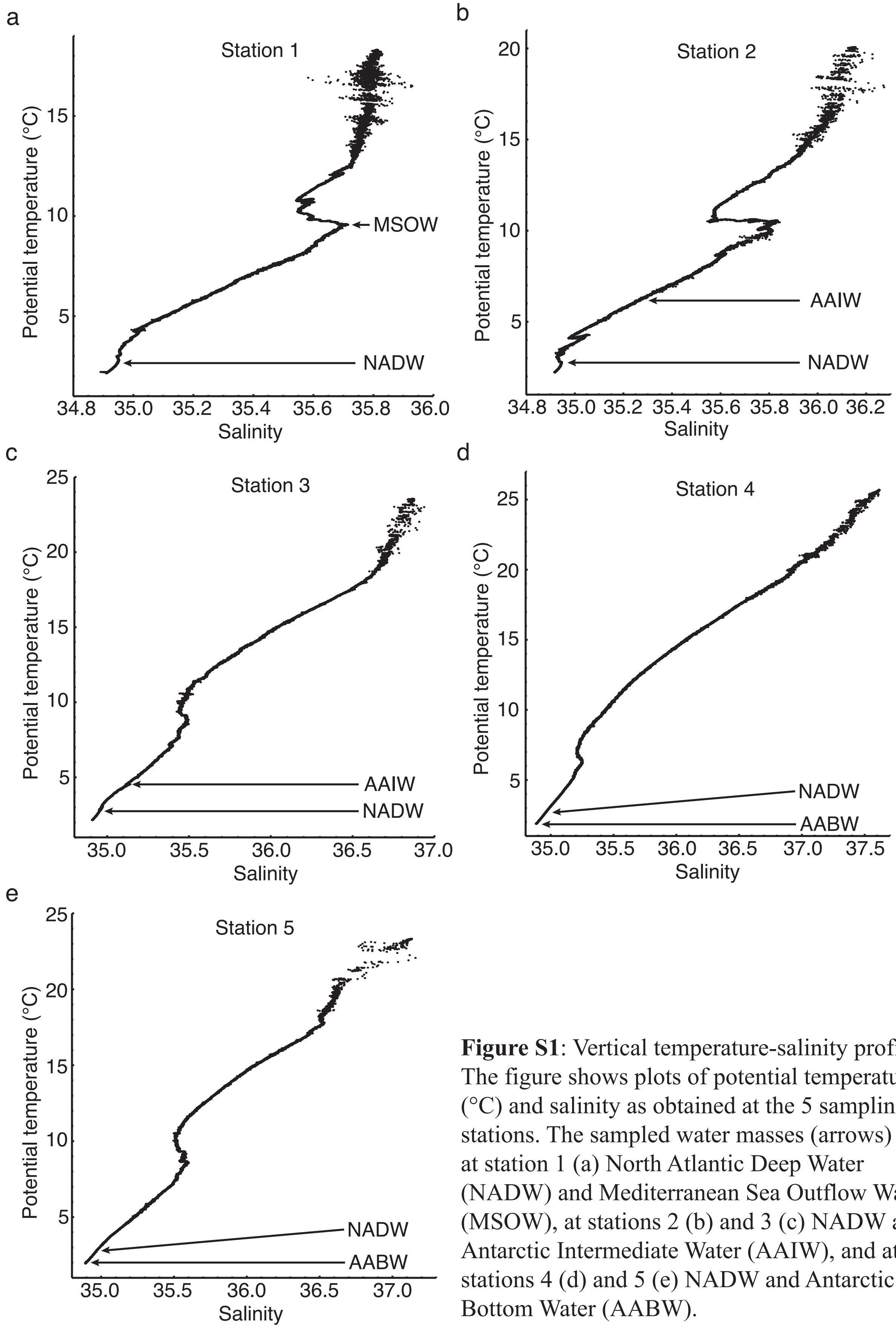

Figure S1: Vertical temperature-salinity profiles. The figure shows plots of potential temperature (°C) and salinity as obtained at the 5 sampling stations. The sampled water masses (arrows) were at station 1 (a) North Atlantic Deep Water (NADW) and Mediterranean Sea Outflow Water (MSOW), at stations 2 (b) and 3 (c) NADW and Antarctic Intermediate Water (AAIW), and at stations 4 (d) and 5 (e) NADW and Antarctic Bottom Water (AABW).

a

	Control 1	Treatment 1	Control 2	Treatment 2
Duplicate 1	P-Water mass 1 in Water mass 1	P-Water mass 1 in Water mass 2	P-Water mass 2 in Water mass 2	P-Water mass 2 in Water mass 1
Duplicate 2	P-Water mass 1 in Water mass 1	P-Water mass 1 in Water mass 2	P-Water mass 2 in Water mass 2	P-Water mass 2 in Water mass 1

b

	Mixing control	Mixing treatment 1	Mixing treatment 2
Duplicate 1	P-Water mass 1 P-Water mass 2 in Water mass 1 Water mass 2	P-Water mass 1 P-Water mass 2 in Water mass 1	P-Water mass 1 P-Water mass 2 in Water mass 2
Duplicate 2	P-Water mass 1 P-Water mass 2 in Water mass 1 Water mass 2	P-Water mass 1 P-Water mass 2 in Water mass 1	P-Water mass 1 P-Water mass 2 in Water mass 2

c

$$\begin{bmatrix} \left[\frac{Treat1Dupl1}{Control1Dupl1}, \frac{Treat1Dupl1}{Control1Dupl2}, \frac{Treat1Dupl2}{Control1Dupl1}, \frac{Treat1Dupl2}{Control1Dupl2}\right] \\ \left[\frac{Treat2Dupl1}{Control2Dupl1}, \frac{Treat2Dupl1}{Control2Dupl2}, \frac{Treat2Dupl2}{Control2Dupl1}, \frac{Treat2Dupl2}{Control2Dupl2}\right] \\ \left[\frac{MixTreat1Dupl1}{MixControlDupl1}, \frac{MixTreat1Dupl1}{MixControlDupl2}, \frac{MixTreat1Dupl2}{MixControlDupl1}, \frac{MixTreat1Dupl2}{MixControlDupl2}\right] \\ \left[\frac{MixTreat2Dupl1}{MixControlDupl1}, \frac{MixTreat2Dupl1}{MixControlDupl2}, \frac{MixTreat2Dupl2}{MixControlDupl1}, \frac{MixTreat2Dupl2}{MixControlDupl2}\right] \end{bmatrix}$$

d

$$\begin{bmatrix} \left[\frac{Leu2}{Leu1}, \frac{Prok2}{Prok1}, \frac{Vir2}{Vir1}\right] \\ \left[\frac{Leu1}{Leu2}, \frac{Prok1}{Prok2}, \frac{Vir1}{Vir2}\right] \\ \left[\frac{1}{2} \times \frac{Leu1}{Leu2}, \frac{1}{2} \times \frac{Prok1}{Prok2}, \frac{1}{2} \times \frac{Vir1}{Vir2}\right] \\ \left[\frac{1}{2} \times \frac{Leu2}{Leu1}, \frac{1}{2} \times \frac{Prok2}{Prok1}, \frac{1}{2} \times \frac{Vir2}{Vir1}\right] \end{bmatrix}$$

e

$$\begin{bmatrix} \left[\frac{Temp2}{Temp1}, \frac{Sal2}{Sal1}\right] \\ \left[\frac{Temp1}{Temp2}, \frac{Sal1}{Sal2}\right] \\ \left[\frac{1}{2} \times \frac{Temp1}{Temp2}, \frac{1}{2} \times \frac{Sal1}{Sal2}\right] \\ \left[\frac{1}{2} \times \frac{Temp2}{Temp1}, \frac{1}{2} \times \frac{Sal2}{Sal1}\right] \end{bmatrix}$$

Figure S2: Experimental design and data treatment. The figure shows the experimental design (a, b) and data treatment of experimental (c) and *in situ* data (d, e) to form the matrices subsequently used in statistical analyses. The prefix "P" (a, b) indicates a prokaryotic concentrate obtained from a specific water mass. Used abbreviations ("X" here corresponds to either "1" or "2" indicating one of two sampled water masses at each of the 5 stations and experiments or the duplicate number): Treatment X-Duplicate X: TreatXDuplX; Control X-Duplicate X: ControlXDuplX; Mixing treatment X-Duplicate X: MixTreatXDuplX; Mixing control X-Duplicate X: MixControlXDuplX; prokaryotic leucine incorporation X: LeuX; prokaryotic abundance X: ProkX; viral abundance X: VirX; temperature: TempX; salinity X: SalX.

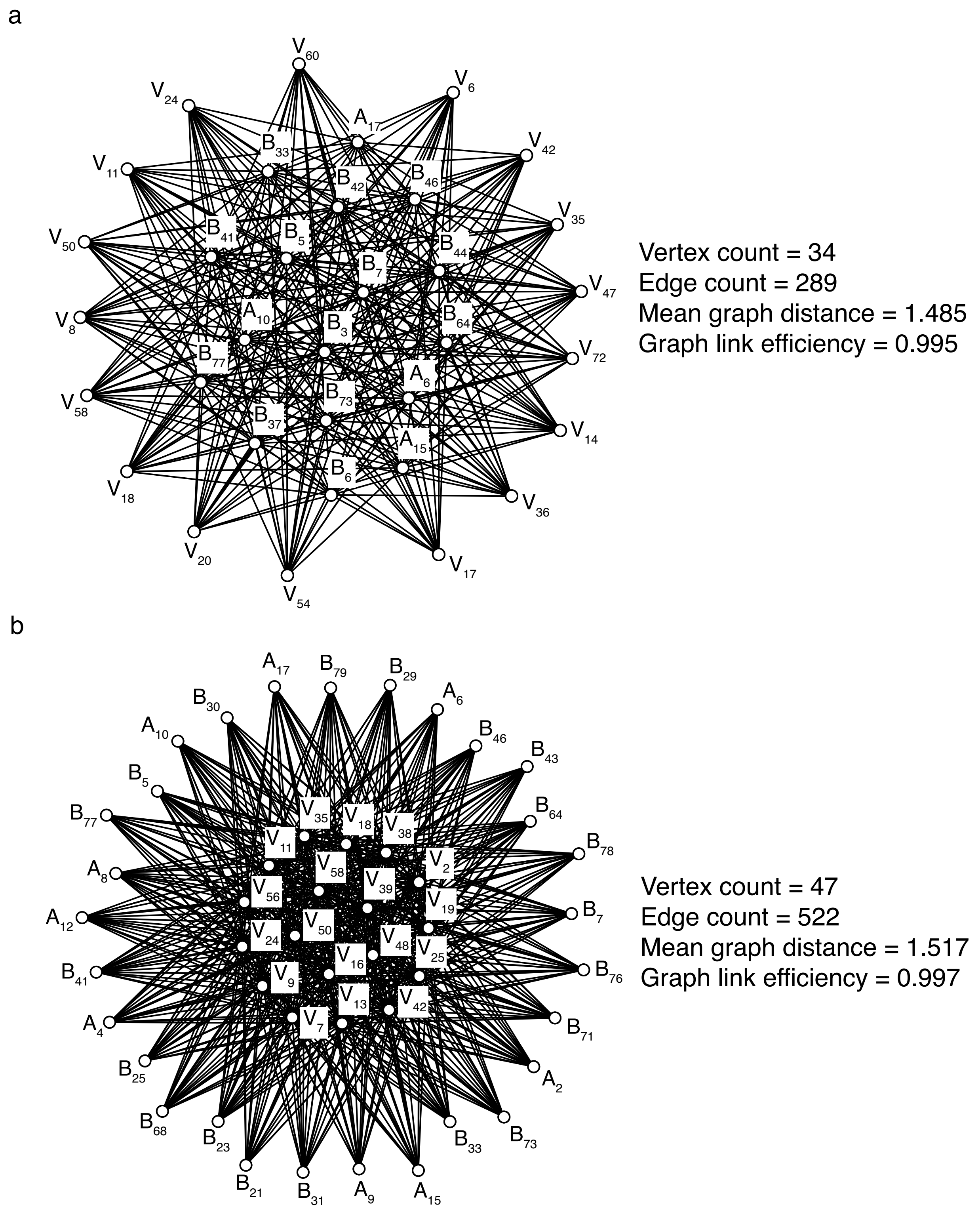

Figure S3: Examples of co-occurrence networks. The figure shows examples of networks based on co-occurrence patterns of prokaryotes with viruses in experiment 1 after 24 h of incubation and also give the corresponding vertex count, edge count, mean graph distance, and graph link efficiency. The networks are from (a) duplicate 1 of the mixture of prokaryotes from North Atlantic Deep Water (NADW) and Mediterranean Sea Outflow Water (MSOW) incubated in MSOW and (b) duplicate 2 of prokaryotes from MSOW incubated in NADW. Archaeal, bacterial, and viral operational taxonomic units are indicated by A_x, B_x, and V_x, respectively.

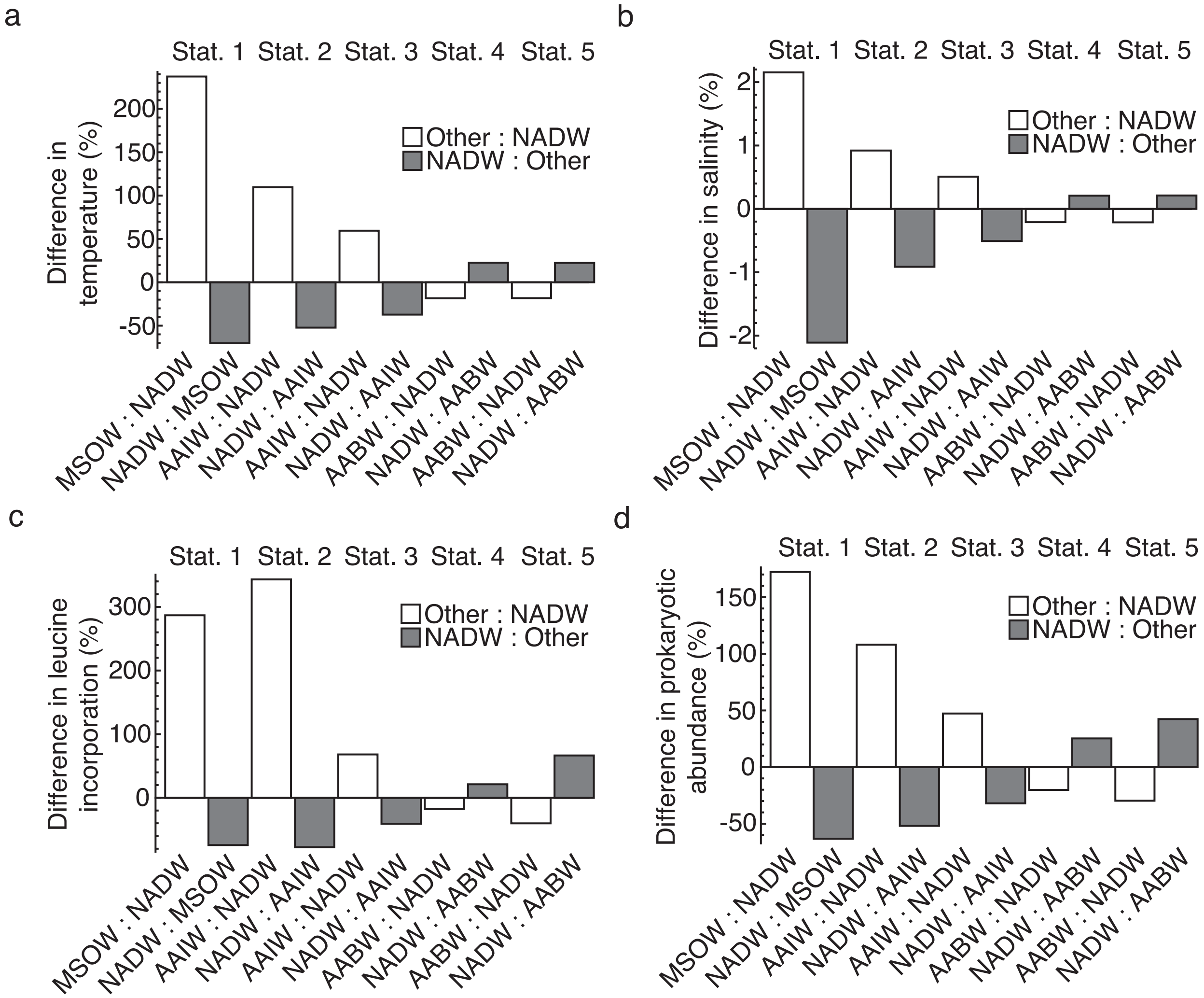

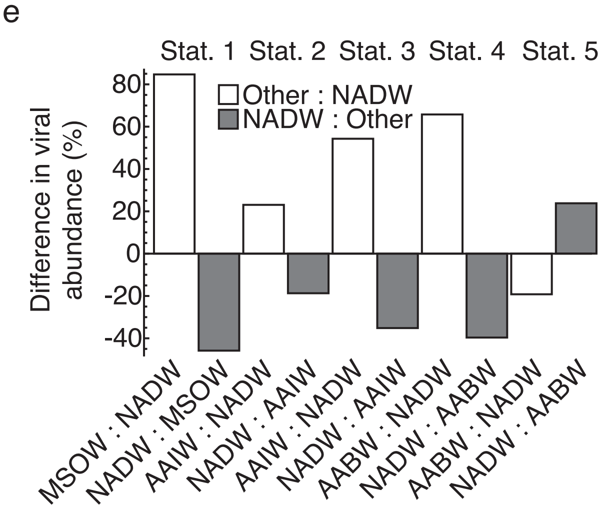

Figure S4: Differences in physico-chemical and biological parameters between water masses. The figure shows differences in (a) salinity, (b) temperature, (c) prokaryotic leucine incorporation, (d) prokaryotic abundance, and (e) viral abundance between North Atlantic Deep Water (NADW), Mediterranean Sea Outflow Water (MSOW), Antarctic Intermediate Water (AAIW), and Antarctic Bottom Water (AABW). Data are given as percent relative to one of two water masses per station (e.g., difference in temperature of 238% for MSOW : NADW reads that temperature in MSOW was 238% higher compared to NADW).

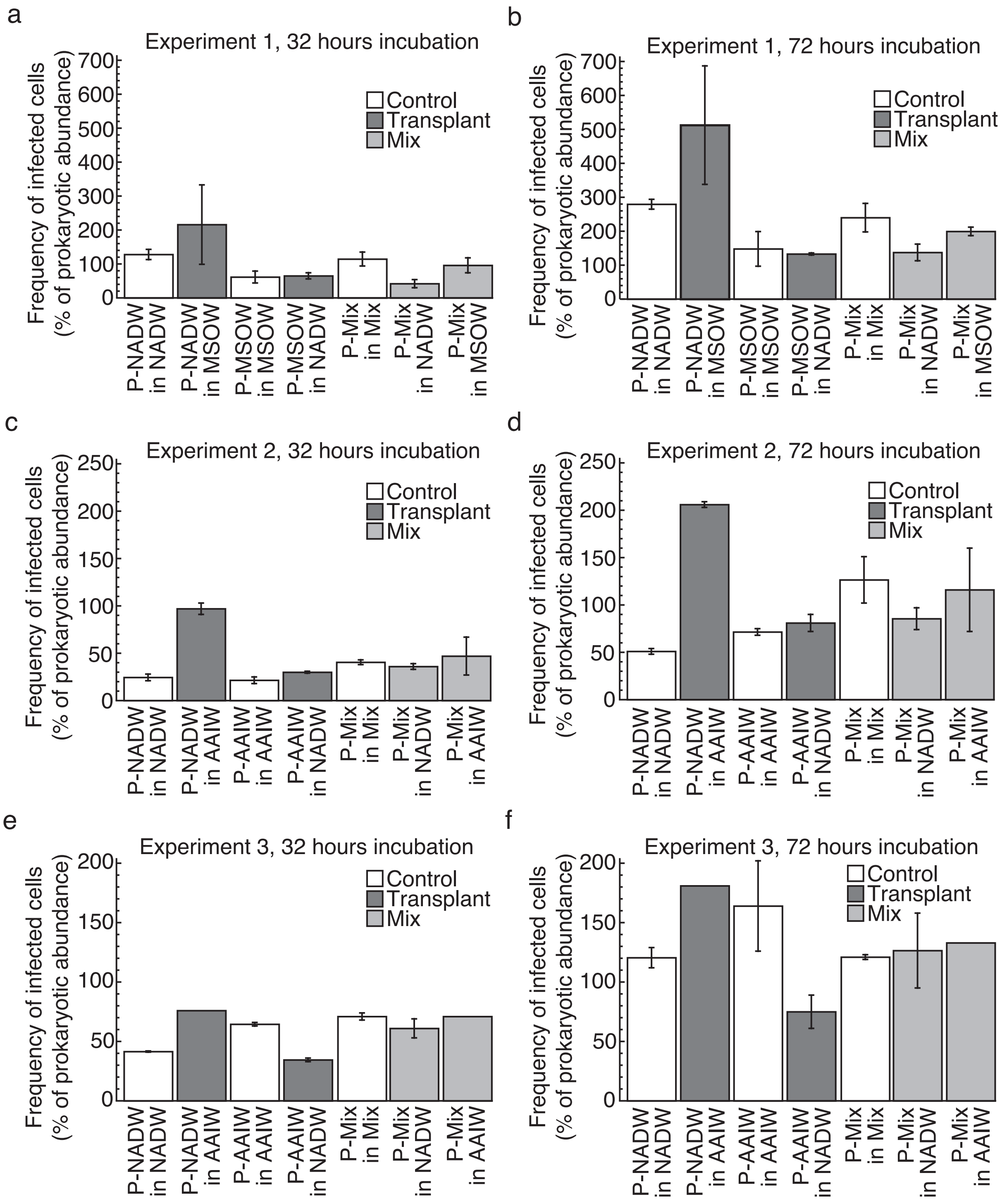

Figure S5: Frequency of infected cells (FIC). The figure shows FIC in percent of prokaryotic abundance at the start of the experiments as estimated in control, transplant, and mixing treatments after 32 h and 72 h of incubation for experiment 1 (a, b), experiment 2 (c, d), experiment 3 (e, f), experiment 4 (g, h), and experiment 5 (i, j). Prokaryotes obtained from a specific water mass are indicated by the prefix "P" followed by either North Atlantic Deep Water (NADW), Mediterranean Sea Outflow Water (MSOW), Antarctic Intermediate Water (AAIW), or Antarctic Bottom Water (AABW). Ultrafiltered water used as incubation medium in the experiments is indicated by the water masses' abbreviation. "P-Mix" represents a mixture of prokaryotes obtained from two water masses and "Mix" a mixture of ultrafiltered water from two water masses. Data are given as average values and error bars represent the range of duplicate incubations.

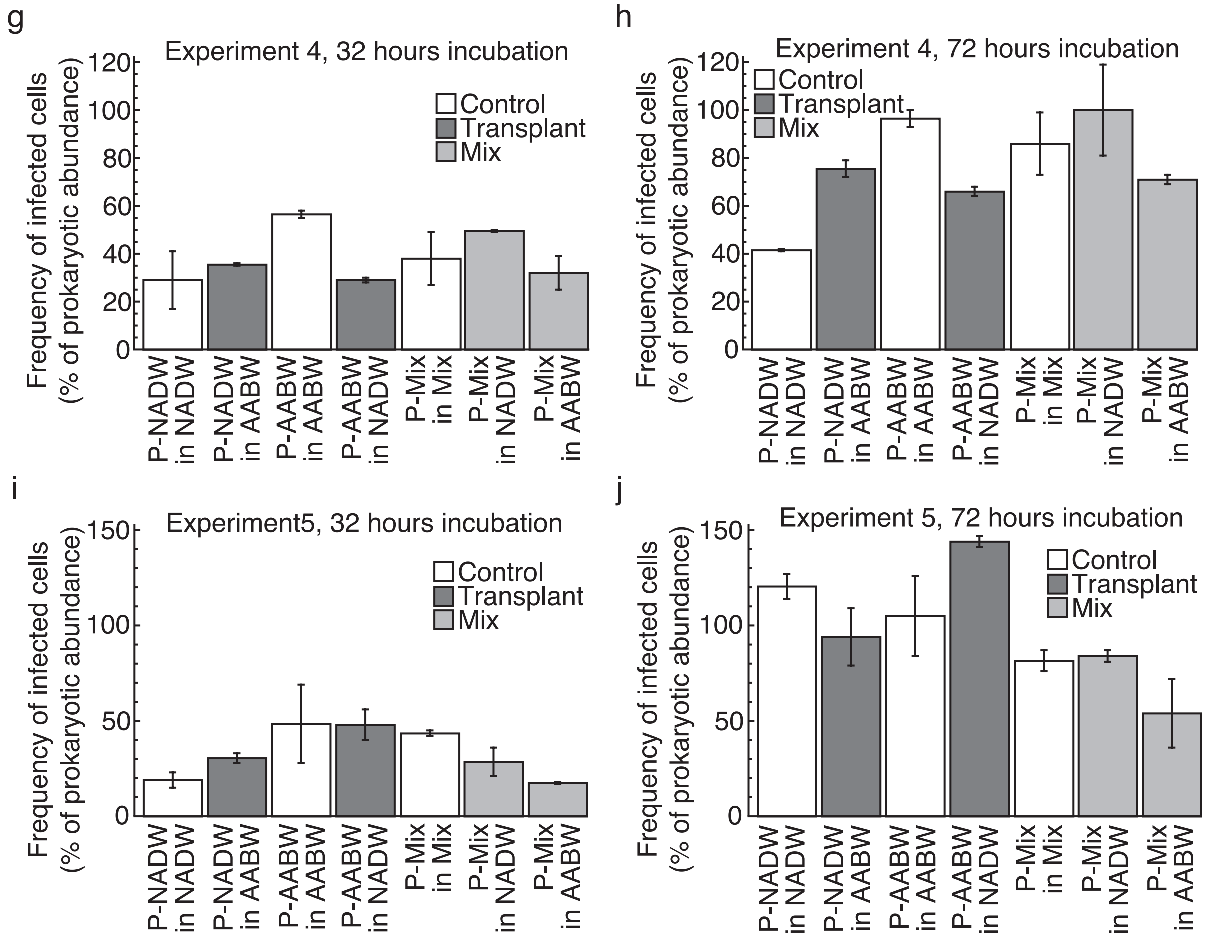

Figure S5 (*continued*)

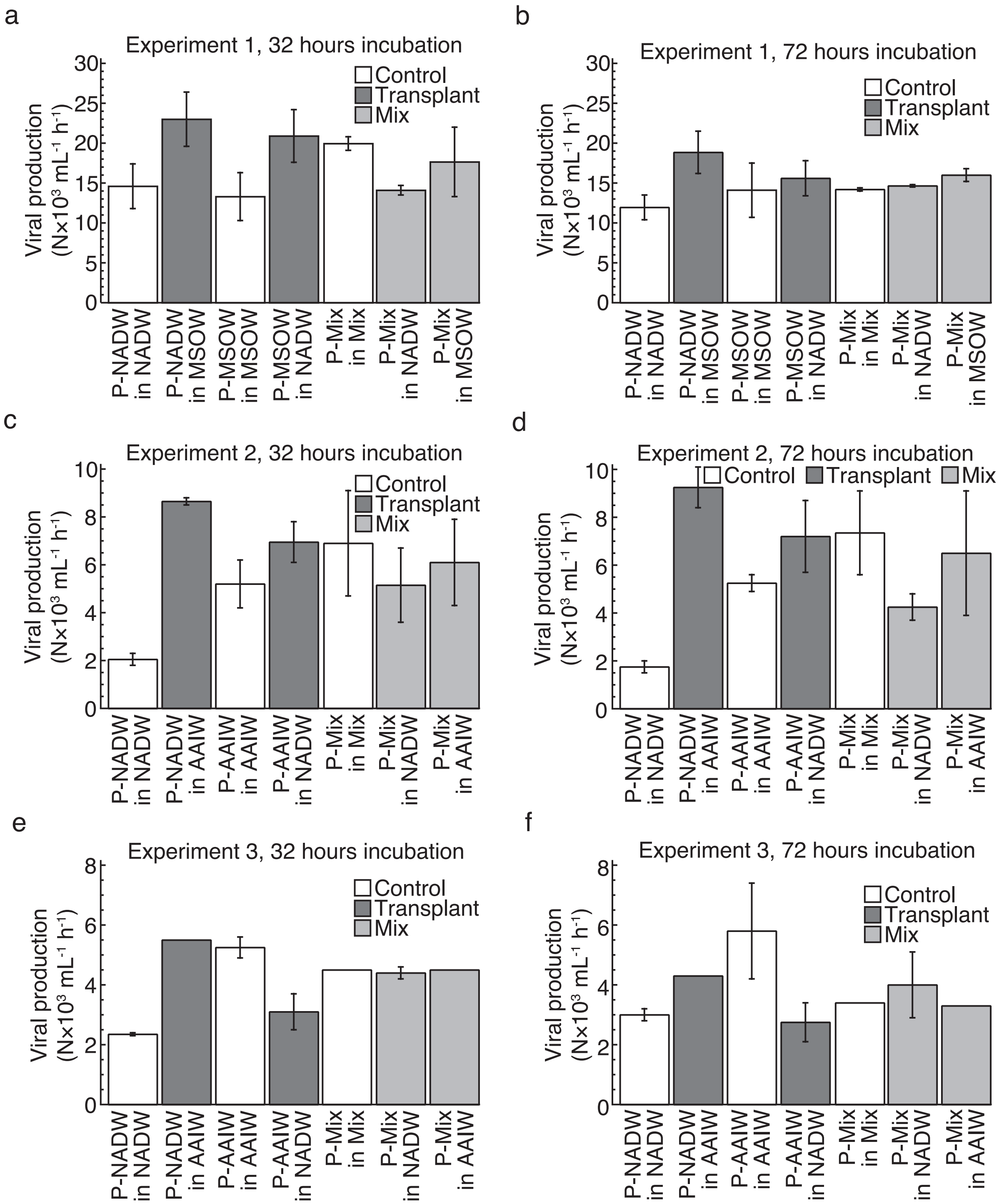

Figure S6: Viral production (VP). The figure shows VP (N×10^{3} mL^{-1} h^{-1}) as estimated in control, transplant, and mixing treatments after 32 h and 72 h of incubation for experiment 1 (a, b), experiment 2 (c, d), experiment 3 (e, f), experiment 4 (g, h), and experiment 5 (i, j). Prokaryotes obtained from a specific water mass are indicated by the prefix "P" followed by either North Atlantic Deep Water (NADW), Mediterranean Sea Outflow Water (MSOW), Antarctic Intermediate Water (AAIW), or Antarctic Bottom Water (AABW). Ultrafiltered water used as incubation medium in the experiments is indicated by the water masses' abbreviation. "P-Mix" represents a mixture of prokaryotes obtained from two water masses and "Mix" a mixture of ultrafiltered water from two water masses. Data are given as average values and error bars represent the range of duplicate incubations.

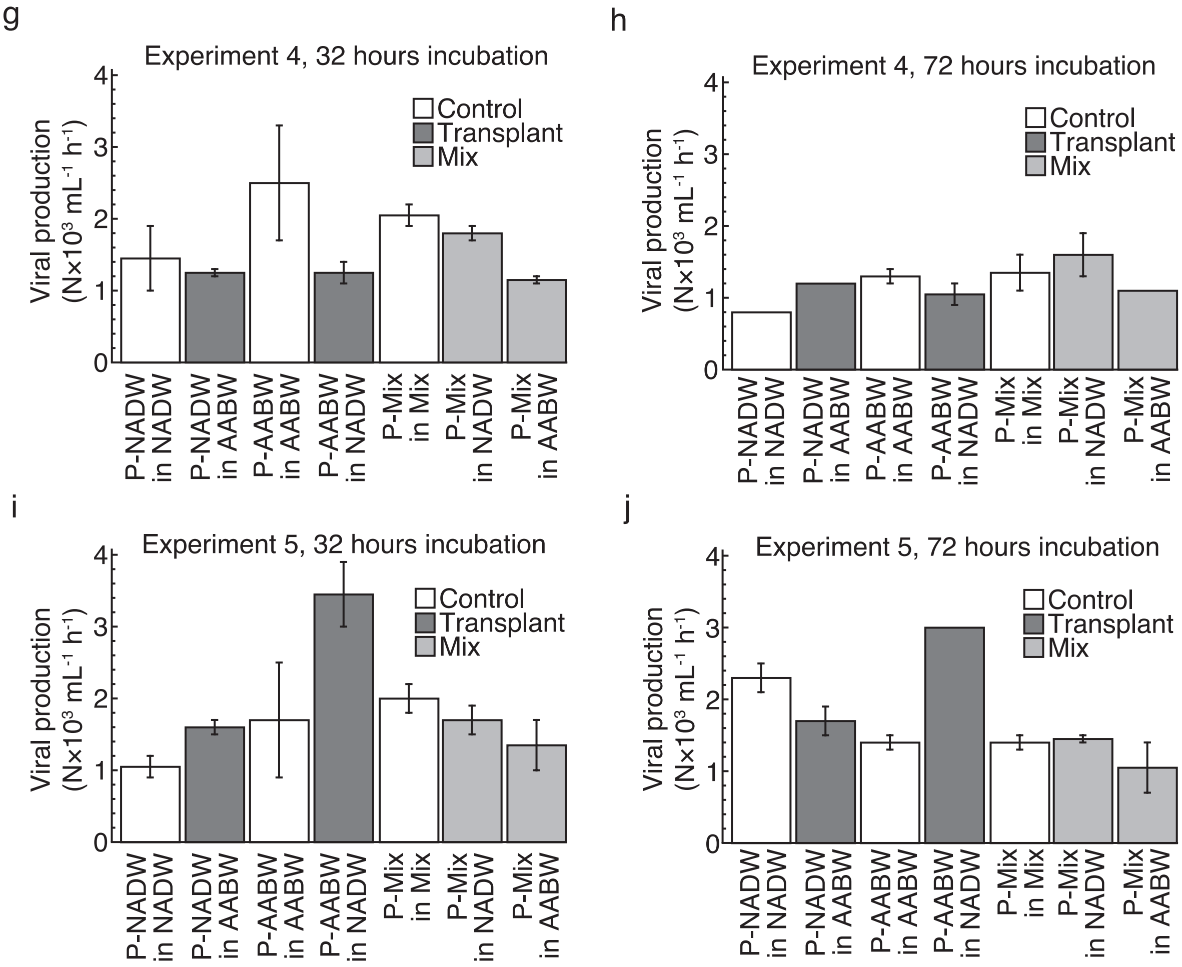

Figure S6 (*continued*)

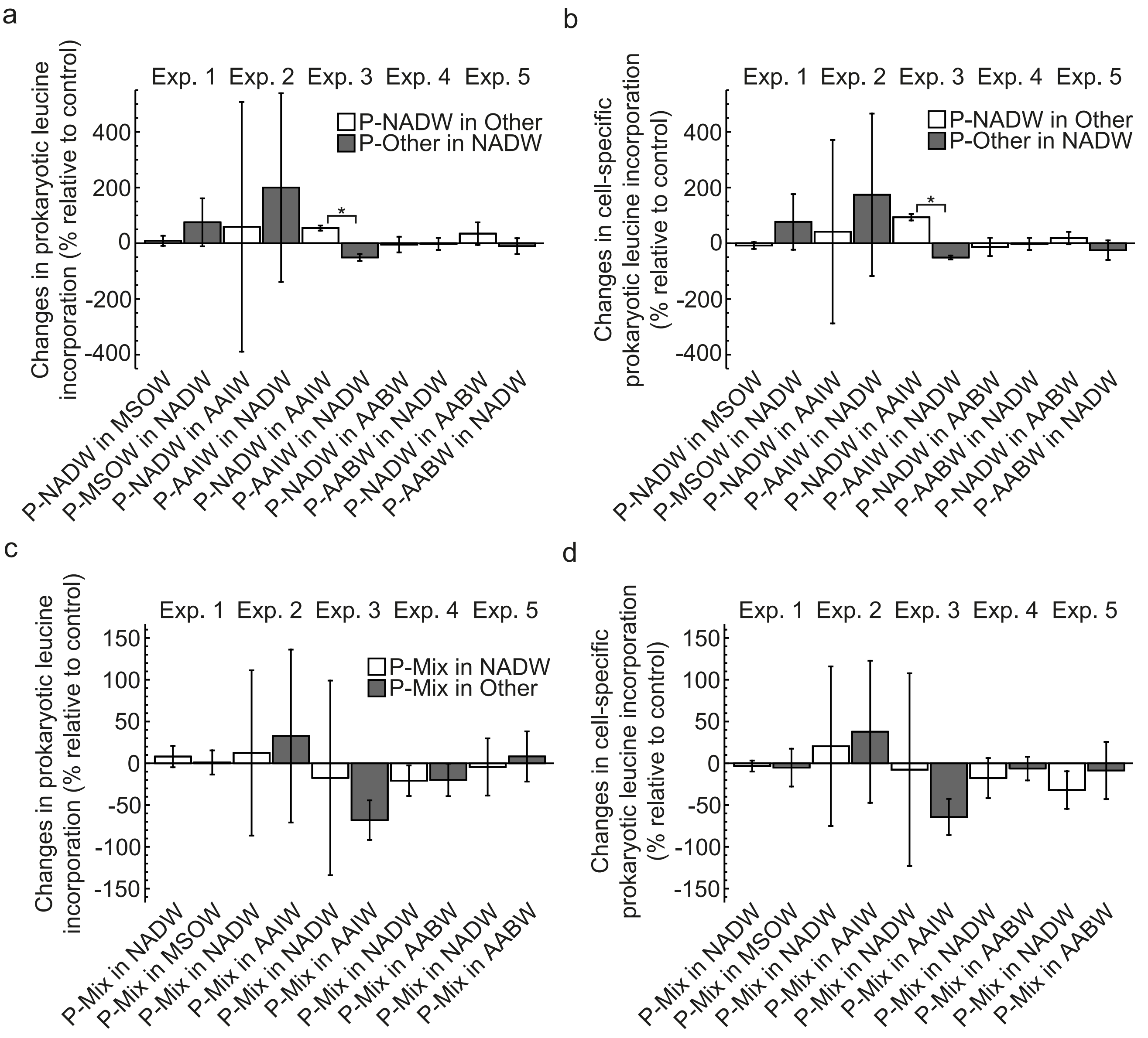

Figure S7: The effect of transplantation on prokaryotic leucine incorporation. The figure shows changes in prokaryotic leucine incorporation (a, c) and cell-specific prokaryotic leucine incorporation (b, d) relative to the control treatments after 72 hours. Prokaryotes obtained from a specific water mass are indicated by the prefix "P" followed by either North Atlantic Deep Water (NADW), Mediterranean Sea Outflow Water (MSOW), Antarctic Intermediate Water (AAIW), or Antarctic Bottom Water (AABW) whereas treatments with prokaryotes obtained from two water masses are indicated by "P-Mix". Ultrafiltered water used as incubation medium in the experiments is indicated by the water masses' abbreviation. Data are given as average values and error bars represent the standard deviation. Brackets with "*" indicate significant differences between the treatments of a specific experiment based on a Mann-Whitney *U* test.

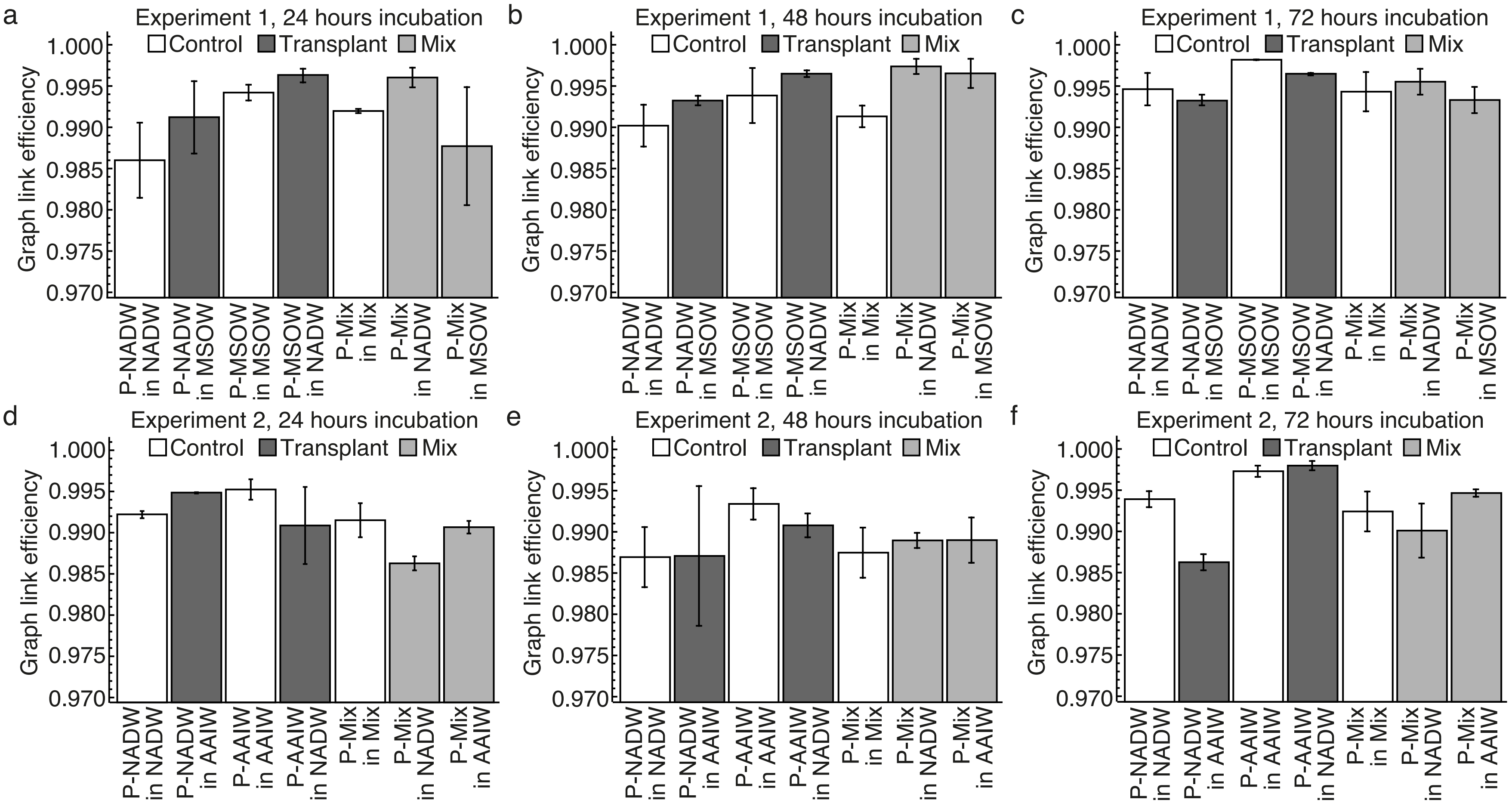

Figure S8: Graph link efficiency. The figure gives the graph link efficiency based on co-occurrence patterns of prokaryotes with viruses translated into networks. The graph link efficiency is presented for control, transplant, and mixing treatments after 24 h, 48 h, and 72 h of incubation for experiment 1 (a–c), experiment 2 (d–f), experiment 3 (g–i), experiment 4 (j–l), and experiment 5 (m–o). Prokaryotes obtained from a specific water mass are indicated by the prefix "P" followed by either North Atlantic Deep Water (NADW), Mediterranean Sea Outflow Water (MSOW), Antarctic Intermediate Water (AAIW), or Antarctic Bottom Water (AABW). Ultrafiltered water used as incubation medium in the experiments is indicated by the water masses' abbreviation. "P-Mix" represents a mixture of prokaryotes obtained from two water masses and "Mix" a mixture of ultrafiltered water from two water masses. Data are given as average values and error bars represent the range of duplicate incubations.

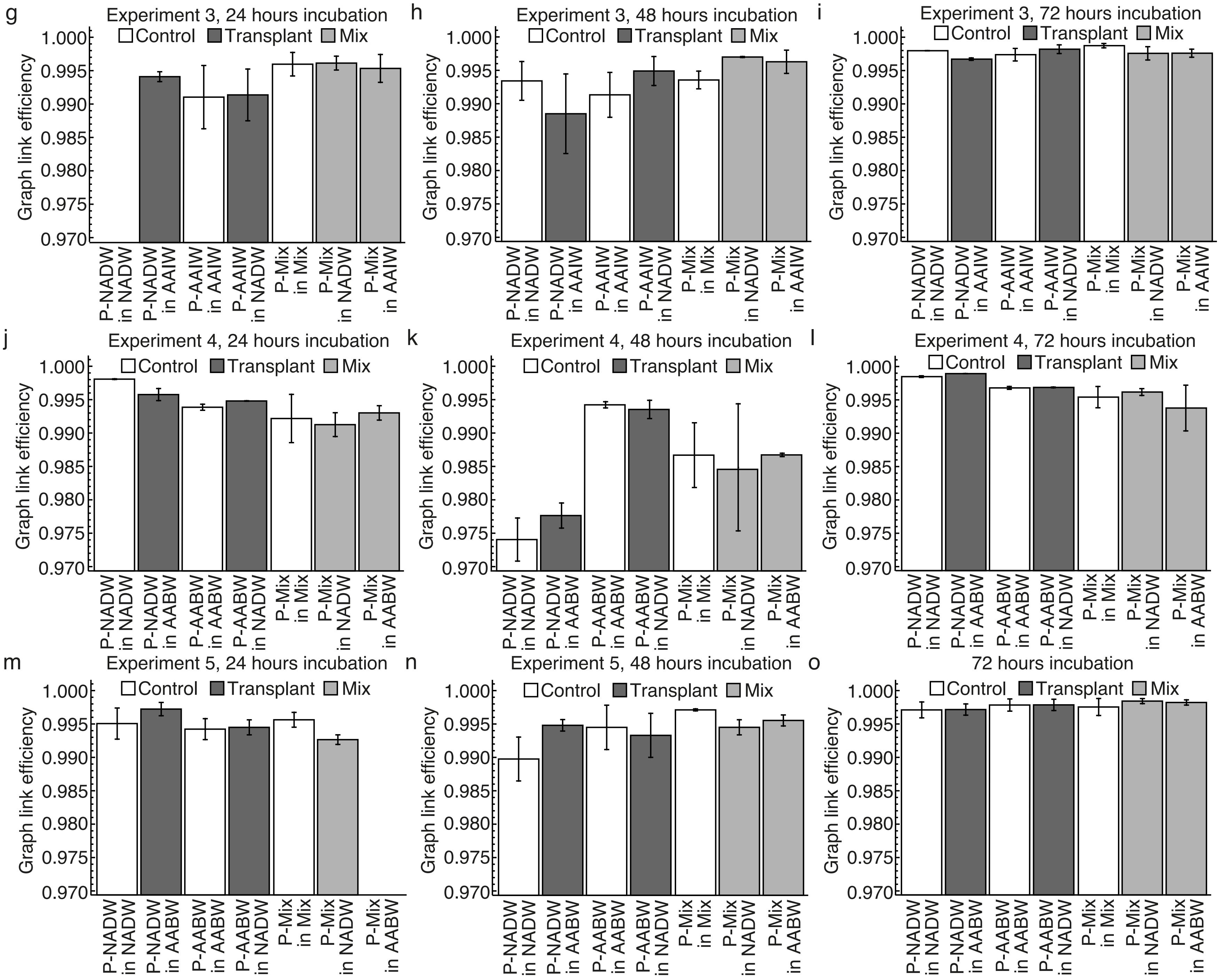

Figure S8 (*continued*)

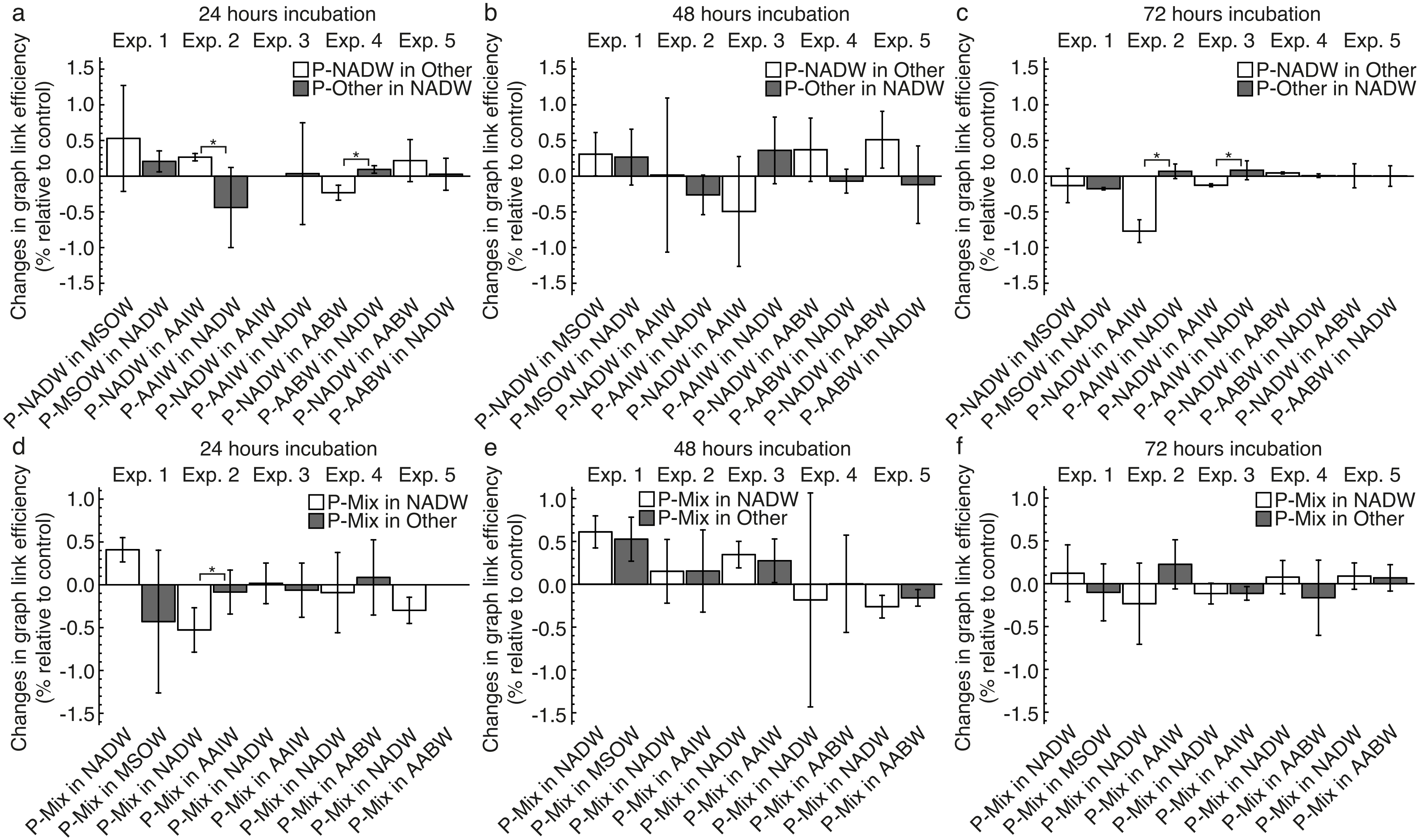

Figure S9: Effects of transplantation on graph link efficiency. The figure shows changes in graph link efficiency based on co-occurrence patterns of prokaryotes with viruses translated into networks in transplantation treatments relative to the control treatments (a–c), and in mixing treatments incubated in one of two water masses relative to mixing treatments incubated in a mixture of both water masses as a control (d–f). Prokaryotes obtained from a specific water mass are indicated by the prefix "P" followed by either North Atlantic Deep Water (NADW), Mediterranean Sea Outflow Water (MSOW), Antarctic Intermediate Water (AAIW), or Antarctic Bottom Water (AABW). Ultrafiltered water used as incubation medium in the experiments is indicated by the water masses' abbreviation. "P-Mix" represents a mixture of prokaryotes obtained from two water masses and "Mix" a mixture of ultrafiltered water from two water masses. Data are presented after 24 h (a, d), 48 h (b, e), and 72 h (c, f) of incubation as average values and error bars represent the standard deviation. Brackets with "*" indicate significant differences between the treatments of a specific experiment based on a Mann-Whitney *U* test.
