# Supplementary material for: Mixing alters the lytic activity of viruses in the dark ocean
Source: Ecology. 2018 Feb 6;99(3):700–13. doi: 10.1002/ecy.2135 (PMC5905300; doi:10.1002/ecy.2135)
Supplement: Supplementary file 3 [file ECY-99-700-s003.pdf]

Mixing alters the lytic activity of viruses in the dark ocean

Christian Winter, Nicole Köstner, Carl-Philip Kruspe, Damaris Urban, Simone Muck, Thomas Reinthaler, Gerhard J. Herndl

Appendix S3

Figure S1: Temporal development of viral abundance in virus dilution incubations

Equation 1: Frequency of infected cells (FIC)

Equation 2: Viral production (VP)

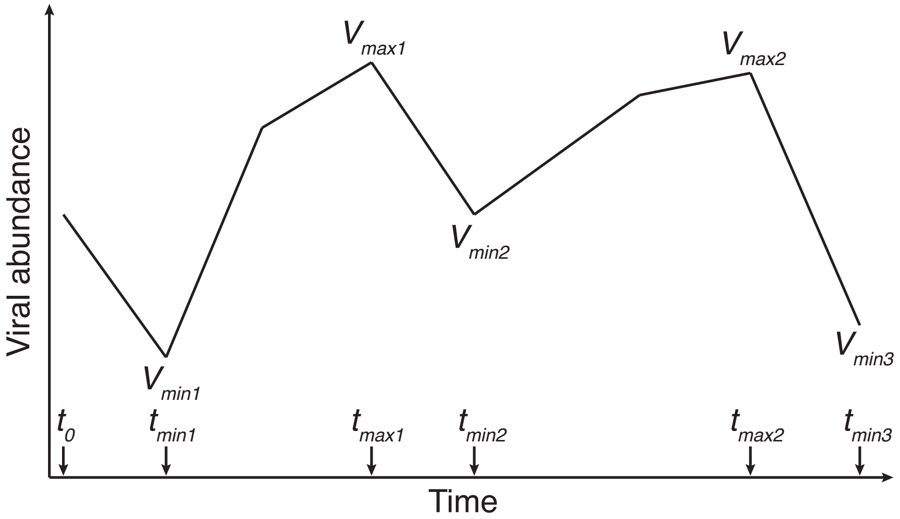

Figure S1: Temporal development of viral abundance in virus dilution incubations. The sketch depicts a typical example of the temporal development of viral abundance commonly obtained from virus dilution incubations. Additionally, the figure depicts local maxima (V_{max1}, V_{max2}) and minima (V_{min1}, V_{min2}, V_{min3}) of viral abundance and the corresponding time points (t_{min1}, t_{max1}, t_{min2}, t_{max2}, t_{min3}) that are used to calculate the frequency of infected cells (FIC, Eq. S1) and viral production (VP, Eq. S2); start of the incubation: t_0.

Equation S1: $$\text{FIC} = \frac{[(V_{max1} - V_{min1}) + (V_{max2} - V_{min2})]}{\text{burst size}} \times \frac{100}{\text{prokaryotic abundance at } t_0}$$

Equation S2: $$\text{VP} = \frac{[(V_{max1} - V_{min1}) + (V_{max2} - V_{min2})]}{(t_{max2} - t_{min1})}$$

The number of maxima and minima in the temporal development of viral abundance may vary so that the equations will have to be adapted accordingly.
