# Supplementary material for: Mixing alters the lytic activity of viruses in the dark ocean
Source: Ecology. 2018 Feb 6;99(3):700–13. doi: 10.1002/ecy.2135 (PMC5905300; doi:10.1002/ecy.2135)
Supplement: Supplementary file 4 [file ECY-99-700-s004.pdf]

Christian Winter, Nicole Köstner, Carl-Philip Kruspe, Damaris Urban, Simone Muck, Thomas Reinthaler, Gerhard J. Herndl. Mixing alters the lytic activity of viruses in the dark ocean. *Ecology*

Data S1

Fingerprinting data obtained from bacterial, archaeal, and viral communities.

Authors

Christian Winter
Department of Limnology and Bio-Oceanography, Center of Ecology, Univ. of Vienna
Althanstrasse 14, 1090 Vienna, Austria
phone: +43 1 4277 76433, email: christian.winter@univie.ac.at

Nicole Köstner
Department of Limnology and Bio-Oceanography, Center of Ecology, Univ. of Vienna
Althanstrasse 14, 1090 Vienna, Austria

Carl-Philip Kruspe
Department of Limnology and Bio-Oceanography, Center of Ecology, Univ. of Vienna
Althanstrasse 14, 1090 Vienna, Austria

Damaris Urban
Department of Limnology and Bio-Oceanography, Center of Ecology, Univ. of Vienna
Althanstrasse 14, 1090 Vienna, Austria

Simone Muck
Department of Limnology and Bio-Oceanography, Center of Ecology, Univ. of Vienna
Althanstrasse 14, 1090 Vienna, Austria

Thomas Reinthaler
Department of Limnology and Bio-Oceanography, Center of Ecology, Univ. of Vienna
Althanstrasse 14, 1090 Vienna, Austria
phone: +43 1 4277 76432, email: thomas.reinthaler@univie.ac.at

Gerhard J. Herndl
Department of Limnology and Bio-Oceanography, Center of Ecology, Univ. of Vienna
Althanstrasse 14, 1090 Vienna, Austria
phone: +43 1 4277 76431, email: gerhard.herndl@univie.ac.at

File list (files found within DataS1.zip)

exp1archforward.csv
exp1archreverse.csv
exp1bacforward.csv
exp1bacreverse.csv
exp1cra22.csv
exp1opa13.csv
exp2archforward.csv
exp2archreverse.csv
exp2bacforward.csv
exp2bacreverse.csv
exp2cra22.csv
exp2opa13.csv
exp3archforward.csv
exp3archreverse.csv
exp3bacforward.csv
exp3bacreverse.csv
exp3cra22.csv
exp3opa13.csv
exp4archforward.csv
exp4archreverse.csv
exp4bacforward.csv
exp4bacreverse.csv
exp4cra22.csv
exp4opa13.csv

exp5archforward.csv

exp5archreverse.csv

exp5bacforward.csv

exp5bacreverse.csv

exp5cra22.csv

exp5opa13.csv

Description

Valid for all data files: exp: number of experiment, time: time after start of incubations, 0: peak or band not present, 1: peak or band present, \N: placeholder for missing data in the SQL language.

Experiment 1 treatments: a1, b1: P-MSOW in MSOW; a2, b2: P-MSOW in NADW; a3, b3: P-NADW in MSOW; a4, b4: P-NADW in NADW; a5, b5: P-Mixture in MSOW; a6, b6: P-Mixture in NADW; a7, b7: P-Mixture in Mixture.

`exp1archforward.csv` - archaeal fingerprinting data obtained with the forward primer from experiment 1.

`exp1archreverse.csv` - archaeal fingerprinting data obtained with the reverse primer from experiment 1.

`exp1bacforward.csv` - bacterial fingerprinting data obtained with the forward primer from experiment 1.

`exp1bacreverse.csv` - bacterial fingerprinting data obtained with the reverse primer from experiment 1.

`exp1cra22.csv` - viral fingerprinting data obtained with the primer CRA22 from experiment 1.

`exp1opa13.csv` - viral fingerprinting data obtained with the primer OPA13 from experiment 1.

Experiment 2 treatments: a1, b1: P-AAIW in AAIW; a2, b2: P-AAIW in NADW; a3, b3: P-NADW in AAIW; a4, b4: P-NADW in NADW; a5, b5: P-Mixture in AAIW; a6, b6: P-Mixture in NADW; a7, b7: P-Mixture in Mixture.

`exp2archforward.csv` - archaeal fingerprinting data obtained with the forward primer from experiment 2.

`exp2archreverse.csv` - archaeal fingerprinting data obtained with the reverse primer from experiment 2.

`exp2bacforward.csv` - bacterial fingerprinting data obtained with the forward primer from experiment 2.

`exp2bacreverse.csv` - bacterial fingerprinting data obtained with the reverse primer from experiment 2.

`exp2cra22.csv` - viral fingerprinting data obtained with the primer CRA22 from experiment 2.

`exp2opa13.csv` - viral fingerprinting data obtained with the primer OPA13 from experiment 2.

Experiment 3 treatments: a1, b1: P-AAIW in AAIW; a2, b2: P-AAIW in NADW; a3, b3: P-NADW in AAIW; a4, b4: P-NADW in NADW; a5, b5: P-Mixture in AAIW; a6, b6: P-Mixture in NADW; a7, b7: P-Mixture in Mixture.

`exp3archforward.csv` - archaeal fingerprinting data obtained with the forward primer from experiment 3.

`exp3archreverse.csv` - archaeal fingerprinting data obtained with the reverse primer from experiment 3.

`exp3bacforward.csv` - bacterial fingerprinting data obtained with the forward primer from experiment 3.

`exp3bacreverse.csv` - bacterial fingerprinting data obtained with the reverse primer from experiment 3.

`exp3cra22.csv` - viral fingerprinting data obtained with the primer CRA22 from experiment 3.

`exp3opa13.csv` - viral fingerprinting data obtained with the primer OPA13 from experiment 3.

Experiment 4 treatments: a1, b1: P-NADW in NADW; a2, b2: P-NADW in AABW; a3, b3: P-AABW in NADW; a4, b4: P-AABW in AABW; a5, b5: P-Mixture in NADW; a6, b6: P-Mixture in AABW; a7, b7: P-Mixture in Mixture.

`exp4archforward.csv` - archaeal fingerprinting data obtained with the forward primer from experiment 4.

`exp4archreverse.csv` - archaeal fingerprinting data obtained with the reverse primer from experiment 4.

`exp4bacforward.csv` - bacterial fingerprinting data obtained with the forward primer from experiment 4.

`exp4bacreverse.csv` - bacterial fingerprinting data obtained with the reverse primer from experiment 4.

`exp4cra22.csv` - viral fingerprinting data obtained with the primer CRA22 from experiment 4.

`exp4opa13.csv` - viral fingerprinting data obtained with the primer OPA13 from experiment 4.

Experiment 5 treatments: a1, b1: P-NADW in NADW; a2, b2: P-NADW in AABW; a3, b3: P-AABW in NADW; a4, b4: P-AABW in AABW; a5, b5: P-Mixture in NADW; a6, b6: P-Mixture in AABW; a7, b7: P-Mixture in Mixture.

`exp5archforward.csv` - archaeal fingerprinting data obtained with the forward primer from experiment 5.

`exp5archreverse.csv` - archaeal fingerprinting data obtained with the reverse primer from experiment 5.

`exp5bacforward.csv` - bacterial fingerprinting data obtained with the forward primer rom experiment 5.

`exp5bacreverse.csv` - bacterial fingerprinting data obtained with the reverse primer from experiment 5.

`exp5cra22.csv` - viral fingerprinting data obtained with the primer CRA22 from experiment 5.

`exp5opa13.csv` - viral fingerprinting data obtained with the primer OPA13 from
